# Polymerization of *Bacillus subtilis* MreB on a lipid membrane reveals lateral co-polymerization of MreB paralogs and strong effects of cations on filament formation

**DOI:** 10.1186/s12860-020-00319-5

**Published:** 2020-11-04

**Authors:** Simon Dersch, Christian Reimold, Joshua Stoll, Hannes Breddermann, Thomas Heimerl, Hervé Joel Defeu Soufo, Peter L. Graumann

**Affiliations:** 1 Centre for Synthetic Microbiology (SYNMIKRO) Hans-Meerwein Strasse 6, Philipps-Universität Marburg, 35032 Marburg, Germany; 2 Fachbereich Chemie, Hans-Meerwein Strasse, Philipps-Universität Marburg, 35032 Marburg, Germany; 3 Institut für Genetik, Zülpicherstr. 47a, 50674 Köln, Germany; 4 Fachbereich Biologie, Karl-von-Frisch-Straße 10, Philipps-Universität Marburg, 35032 Marburg, Germany; 5 Department of Microsystems Engineering – IMTEK, University of Freiburg, 79110 Freiburg, Germany

## Abstract

**Background:**

MreB is a bacterial ortholog of actin and forms mobile filaments underneath the cell membrane, perpendicular to the long axis of the cell, which play a crucial role for cell shape maintenance. We wished to visualize *Bacillus subtilis* MreB in vitro and therefore established a protocol to obtain monomeric protein, which could be polymerized on a planar membrane system, or associated with large membrane vesicles.

**Results:**

Using a planar membrane system and electron microscopy, we show that *Bacillus subtilis* MreB forms bundles of filaments, which can branch and fuse, with an average width of 70 nm. Fluorescence microscopy of non-polymerized YFP-MreB, CFP-Mbl and mCherry-MreBH proteins showed uniform binding to the membrane, suggesting that 2D diffusion along the membrane could facilitate filament formation. After addition of divalent magnesium and calcium ions, all three proteins formed highly disordered sheets of filaments that could split up or merge, such that at high protein concentration, MreB and its paralogs generated a network of filaments extending away from the membrane. Filament formation was positively affected by divalent ions and negatively by monovalent ions. YFP-MreB or CFP-Mbl also formed filaments between two adjacent membranes, which frequently has a curved appearance. New MreB, Mbl or MreBH monomers could add to the lateral side of preexisting filaments, and MreB paralogs co-polymerized, indicating direct lateral interaction between MreB paralogs.

**Conclusions:**

Our data show that *B. subtilis* MreB paralogs do not easily form ordered filaments in vitro, possibly due to extensive lateral contacts, but can co-polymerise. Monomeric MreB, Mbl and MreBH uniformly bind to a membrane, and form irregular and frequently split up filamentous structures, facilitated by the addition of divalent ions, and counteracted by monovalent ions, suggesting that intracellular potassium levels may be one important factor to counteract extensive filament formation and filament splitting in vivo.

**Supplementary information:**

**Supplementary information** accompanies this paper at 10.1186/s12860-020-00319-5.

## Background

Cell morphology can greatly vary in all cells, providing evolutionary advantages and adaptation to special niches. In spite of the importance of cell shape for bacterial physiology, it is still unclear how the shape of the peptidoglycan cell wall, which dictates cell architecture, is generated at the molecular level. Actin-like MreB protein and its orthologs are key players in this process.

In Eukaryotes, cytoskeletal elements facilitate coordinated functions in multiple cellular processes, which depend on their characteristic properties to form networks of filaments. Besides microtubules and intermediate filaments, actin filaments (filamentous/F-actin) are the most abundant cytoskeletal structures and key organizers of cell morphology, cytokinesis, cellular motility and intracellular transport [1, 2]. The filaments consist of two protofilaments that are arranged as a right-handed double helix [3]. The dynamics of this structure are based on a polar growth at steady state with a net polymerization on one end (plus end) and depolymerization on the other end (minus end), giving rise to a treadmilling-like movement of subunits within the filament [4]. Similar dynamics have been found for other actin-like proteins [5]. Monomeric actin (globular/G-actin) consists of four subdomains with five highly conserved motifs that enclose the nucleotide-binding site as the central core. Homologous proteins harboring these conserved motifs are classified as members of the actin superfamily and are present in all domains of life with highly divergent functions [6–9].

Among the members of this protein family, MreB is one of the most widely conserved prokaryotic actin-homologs. In 2001, crystallization of *Thermotoga maritima* MreB (*Tm*MreB) revealed that the overall size and the three-dimensional structure of monomeric MreB closely resemble those of G-actin, although the sequence identity is limited to around 15% [10]. This structural resemblance also includes the polymeric forms. Crystals containing protofilaments of *Tm*MreB and *Caulobacter crescentus* MreB (*Cc*MreB) revealed that their architectures coincide with that of the actin protofilaments with respect to the polar orientation of the subunits and their longitudinal interface [11]. An additional similarity to actin emerged from electron microcopy, which resolved *Tm*MreB and *Cc*MreB filaments as pairs of protofilaments. However, the protofilaments are straight with an antiparallel orientation to each other and are not twisted and oriented in a parallel fashion as shown for F-actin [10, 11]. The formation of antiparallel protofilaments could also be proven for *Ec*MreB filaments in vitro pointing to a unique feature of MreB within the actin superfamily [11].

MreB is an essential protein in many bacteria with non-spherical morphology and a key determinant of the cell diameter during cell elongation. Depletion of MreB, deletion of its encoding gene (*mreB*), or disruption of MreB structures by A22 (S-[3,4-dichlorobenzyl] isothiourea) causes an arrest in cell wall elongation. Subsequently, cells adopt a spherical morphology and are prone to lysis [12–16]. Studies over the last decade gradually identified interaction and co-localization of MreB with cell wall enzymes in several species suggesting a function of MreB as a spatial organizer of the lateral cell wall synthesis machinery [17]. The morphogenic protein was also shown to be required for cell polarity [18–21] and was reported to have an impact in the coordination of chromosome segregation [12, 22–24], though not unanimously approved [25–28].

Super-resolution microscopy revealed that in the Gram-positive model bacterium *Bacillus subtilis*, MreB (*Bs*MreB) and its paralogs Mbl and MreBH assemble into discontinuous filaments of variable length (average length 1.7 μm) underneath the lateral membrane, which is also true for the single MreB protein present in *E. coli*. The filaments predominantly move perpendicular to the longitudinal axis with a minor fraction exhibiting a maximum tilt of up to 40° [29, 30]. Recent studies suggest a passive movement of the filaments driven by the catalytic energy of the coupled cell wall synthetic complexes [31–33], which contrasts the active, treadmilling-based (or myosin-driven) movement of F-actin. When heterologously co-expressed in the eukaryotic Schneider S2 cell line and in *Escherichia coli*, the three MreB paralogs of *B. subtilis* co-polymerize into a single filament [34, 35], an arrangement that recalls the subcellular localization pattern of the proteins in the host bacterium *B. subtilis* [36, 37]. The mixed filaments localized exclusively at the membrane in the heterologous expression systems [34, 35]. This intrinsic membrane affinity, also shown for MreB from different organisms including *T. maritima*, *E. coli* [38] and *Caulobacter crescentus* [11], revealed another characteristic trait of MreB as compared to its eukaryotic counterpart. MreB proteins of Gram-positive bacteria are predicted to associate with the membrane via a hydrophobic loop while those of Gram-negative bacteria require an additionally contact via an N-terminal amphipathic helix. This second contact appears to confer the majority of the energy required for membrane binding [38].

Polymerization of MreB proteins in vitro has been investigated by dynamic light scattering (DLS) analysis and was shown to be affected by cations. K^+^ as a monovalent cation has an inhibitory effect on the polymerization of *Tm*MreB, *Bs*MreB and *Chlamydophila pneumonia* MreB (*Cp*MreB), whereas Mg^2+^ as a divalent cation stimulates - and in case of *Bs*MreB - induces the assembly [39–41]. However, MreB proteins are notoriously difficult to purify and tend to quickly aggregate. To overcome this limitation, we established a protocol to obtain monomeric *B. subtilis* MreB, Mbl and MreBH, with and without fluorescence tags. We visualized MreB filaments on a planar membrane, using both electron microscopy and fluorescence imaging. While polymerization could be induced as was shown in the DLS studies, MreB paralogs did not form ordered linear filaments in vitro, as would have been expected from in vivo filament formation, indicating the importance of additional regulatory mechanisms in the cell. We were still able to draw conclusions on the mode of filament formation in vitro, including membrane-affinity of monomers, addition of MreB paralogs to preexisting filaments, and co-polymerization, indicating that lateral contacts exist between MreB paralogs.

## Results

### Purification of monomeric MreB

**Fig. 1:**
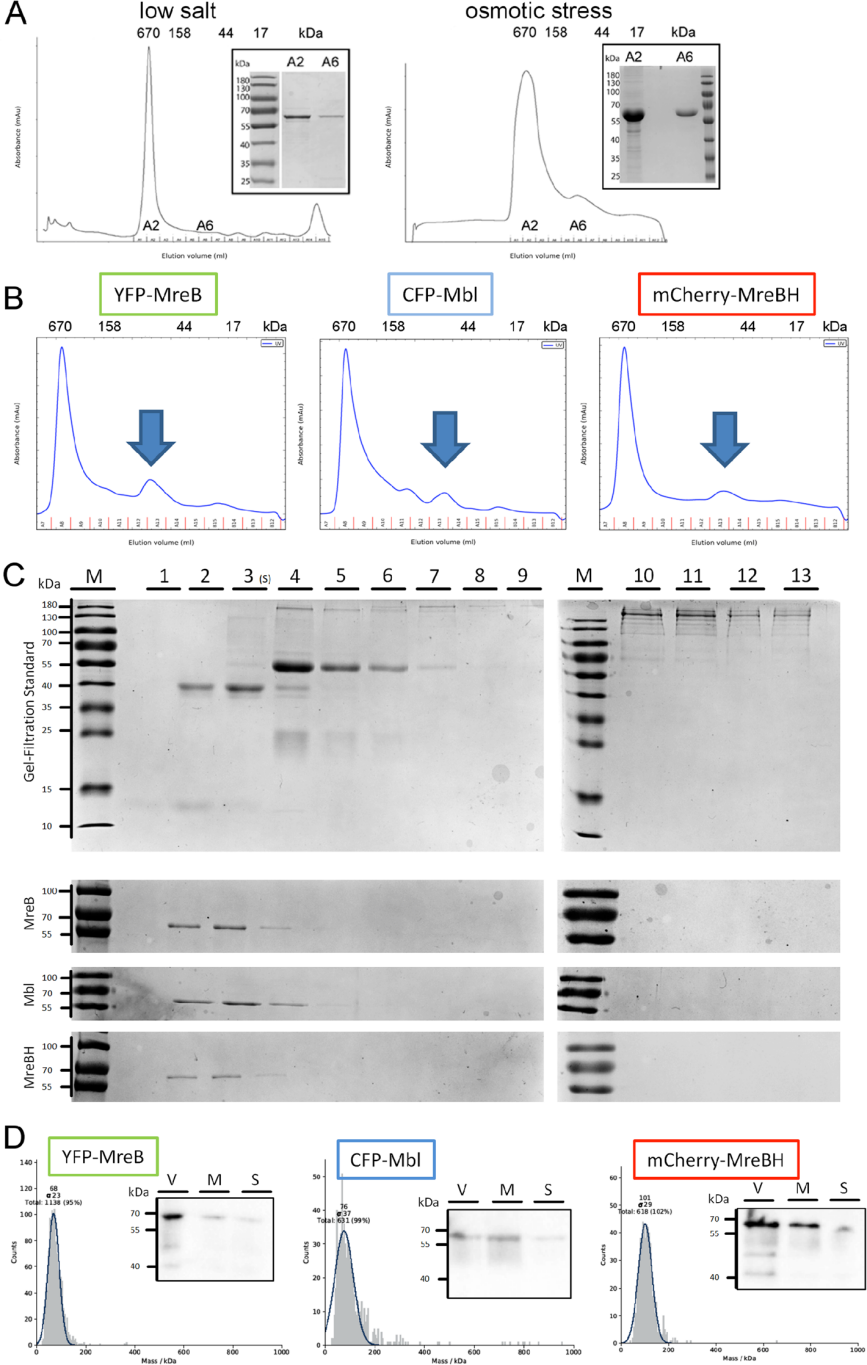
**a** Gel filtration of YFP-MreB after streptactin purification and expression under low salt (100 mM NaCl) or under osmotic stress (expression overnight, addition of 500 mM sorbitol and 1 mM betaine). Molecular standards are shown above the elution peaks. Fraction A2 = void volume, fraction A6 = monomeric YFP-MreB, as shown in the SDS-PAGE inserts (Purification buffer: 100 mM Tris HCl, 300 mM NaCl, 1 mM EDTA, 0.2 mM ATP, 5% Glycerol pH 7.5). **b** Monomer peaks of YFP-MreB, CFP-Mbl, mCherry-MreBH obtained under osmotic stress conditions (indicated by arrows), as outlined previously. **c** Sucrose gradient (5–15%) of isolated monomer peaks of YFP-MreB, CFP-Mbl, mCherry-MreBH. Biorad gel-filtration standard was used as a reference. (Marker proteins appearing in lane 1: Myoglobin, 2: Ovalbumin, 4: Gamma-Globulin, 10: Thyroglobulin; YFP-MreB, CFP-Mbl, mCherry-MreB appear in lane 2). **d** Mass photometric analysis of isolated YFP-MreB, CFP-Mbl, mCherry-MreBH monomers and embedded western blot with specific antibodies against the respective protein (V: void volume, M: isolated monomer peak, S: after dialysis to low salt polymerization buffer)

MreB has been purified under various ionic and buffer conditions, and is known to be prone to spontaneous polymerization or aggregation. Addition of magnesium chloride can efficiently induce filament formation of MreB from various bacteria [10, 11, 39–41], and YFP-MreB from *B. subtilis* polymerizes as efficiently as non-fused MreB [42]. GFP-MreB, GFP-Mbl and GFP-MreBH have been shown to be able to functionally replace wild type proteins in vivo [29, 31, 32, 36]. MreB from *C. crescentus* and *T. maritima* have recently been purified and imaged on vesicles or on a flat membrane, where they form antiparallel double filaments as smallest unit [11]. We wished to obtain a better understanding of MreB filaments from a Gram-positive bacterium. In order to obtain monomeric Strep-YFP-MreB from *B. subtilis*, we tested various previously described as well as novel buffer and growth conditions, but MreB was predominantly present close to the void volume of the gel filtration columns (Fig. 1a), especially under salt concentrations below 300 mM NaCl, only a small amount of monomeric MreB could be obtained (Fig. 1a). Concentrations of over 300 mM NaCl in the purification buffer resulted in flocculent aggregation of MreB (data not shown). Overexpression in media with increased salt or sugar concentrations to induce weak osmotic pressure and the addition of betaine as an osmoprotectant has been shown to reduce the occurrence of aggregation for proteins that are difficult to purify [43]. A combination of overnight expression at low temperatures under weak osmotic pressure with 500 mM sorbitol (plus 1 mM betaine) and purification of the obtained cell pellets using a buffer containing 300 mM salt (Purification buffer: 100 mM Tris HCl, 300 mM NaCl, 1 mM EDTA, 0.2 mM ATP, 5% glycerol pH 7.5) resulted in a peak containing monomeric MreB (Fig. 1a) that could be isolated via size-exclusion chromatography. The protein was dialyzed and stored in low salt storage buffer (5 mM TRIS-HCl, 0.1 mM CaCl_2_, 0.2 mM ATP, pH 7.5).

Although the yield could not be further increased by any additional measure that was tried, the amounts were sufficient for further experiments. Of note, monomeric MreB such as the one from fraction A6 (Fig. 1a, b) remained monomeric for 1 week of storage at 4 °C (data not shown) and retained its polymerization activity. It is likely that conditions in *E. coli* cells favour MreB polymerization, and MreB will only stay monomeric for some time in special buffer conditions. Similar to Strep-YFP-MreB, Strep-CFP-Mbl and Strep-mCherry-MreBH could also be purified as monomers (Fig. 1b). For simplicity, the “Strep” tag is no longer mentioned in the following text. To verify that the isolated fractions were truly monomeric, firstly yields of the respective proteins from the monomer peaks were loaded onto a 5–15% sucrose density gradient and separated via ultracentrifugation. Gel-filtration standard from Biorad was used as a reference (Fig. 1c). The marker proteins were observed in lane 1: Myoglobin (17 kDa), 2: Ovalbumin (44 kDa), 4: Gamma-Globulin (158 kDa), 10: Thyroglobulin (670 kDa), whereas YFP-MreB, CFP-Mbl, mCherry-MreB (~ 65 kDa) all appeared starting in lane 2, indicating low molecular mass (Fig. 1c). There were also visible bands in lane 3 and weak bands in lane 4 for the three fluorophore-tagged paralogs, but no visible band for higher molecular masses. To further test if the peaks isolated from gel filtration were truly monomeric, we performed a photometric mass analysis of the proteins (Fig. 1d) [44]. For all three proteins only a single peak was observed. YFP-MreB showed a monomeric peak at 68 ± 23 kDa, CFP-Mbl at 76 ± 37 and mCherry-MreBH at 101 ± 29 (Fig. 1d). Overall, the photometric approach offered high precision, even though the peak for mCherry-MreBH was slightly higher than the expected size of a monomer. Taken together with other data previously detailed, a monomeric form of the three MreB paralogs could be successfully obtained (Fig. 1a-d). 300 mM NaCl during the purification procedure, together with expression under osmotic pressure, appears to be the most stable condition to avoid polymerization of MreB (which likely occurs at lower salt concentration) or aggregation, which we assume to happen at higher salt conditions.

### MreB forms predominantly bundles of filaments in solution

**Fig. 2:**
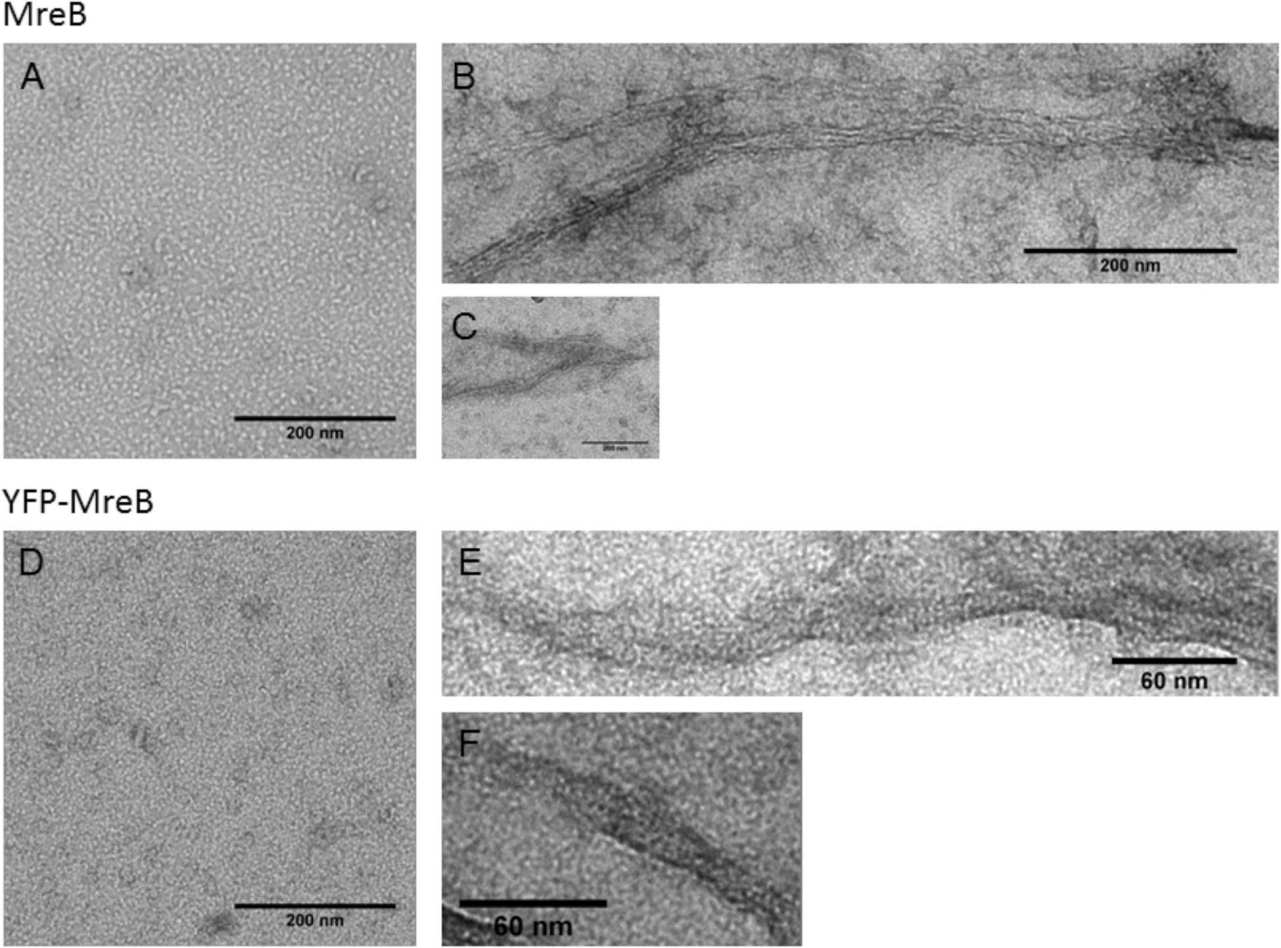
Electron microscopy of negatively-stained MreB solution before and after induction of polymerization. **a** MreB monomers (2 μM) in polymerization buffer (5 mM TRIS-HCl, 0.1 mM CaCl_2_, 0.2 mM ATP, pH 7.5) on EM grid. **b-c** Filaments formed from the same MreB solution as in A, after induction of polymerization with 10 mM MgCl_2_. **d** YFP-MreB monomers (2 μM) in polymerization buffer on EM grid. **e-f** Filaments formed from the same YFP-MreB solution as in D, after induction of polymerization with 10 mM MgCl_2_. Scaling is indicated below the black bars

We used transmission EM and negative staining with uranyl acetate, and visualized monomeric purified MreB and YFP-MreB in low-salt storage buffer (Fig. 2a and d), and polymers after induction of polymerization with 10 mM MgCl_2_ (Fig. 2b/c/e/f). Without the addition of MgCl_2_ no filamentous structures were observed, only small accumulations, likely monomers of MreB and YFP-MreB, were present on the grid (Fig. 2a + d). After induction of polymerization, extended filamentous structures had formed, likely bundles or sheets of individual filaments, which could split up or merge (Fig. 2b, c, e, f). The width of these sheets was in most cases below 200 nm, but occasionally intertwined macrostructures were observed, that could be over 200 nm wide (suppl. Fig. S1B). In some cases these structures appeared to be twisted bundles of protofilaments (suppl. Fig. S1A). Our analyses did not reach a resolution allowing to identify single filaments, as in earlier reports the minimal unit of MreB filaments was shown to consist of two (anti-) parallel protofilaments [10, 11]. Importantly, filaments formed by YFP-MreB (Fig. 2e/f) were visually similar to those of MreB (Fig. 2b/c). Interestingly, most structures were highly irregular, such that many filamentous structures had an overall fuzzy appearance (suppl. Fig. S1A-D). Although these analyses are in general agreement with our measurements of a preferred width of YFP-MreB filaments of 75 nm in vivo [29], we do not believe that the observed structures are close matches of in vivo filaments, which have a much smoother appearance [29, 30]. Nevertheless, the observed structures appear to be filamentous, and thus disordered structures rather than aggregates of MreB monomers. We interpret our findings as consequences of MreB polymerizing away from the membrane, and of the absence of regulatory mechanisms and cellular interactors such as RodZ [45–47] and EF-Tu [42].

### MreB monomers have membrane affinity and form extended filaments after addition of magnesium or of calcium ions

**Fig. 3:**
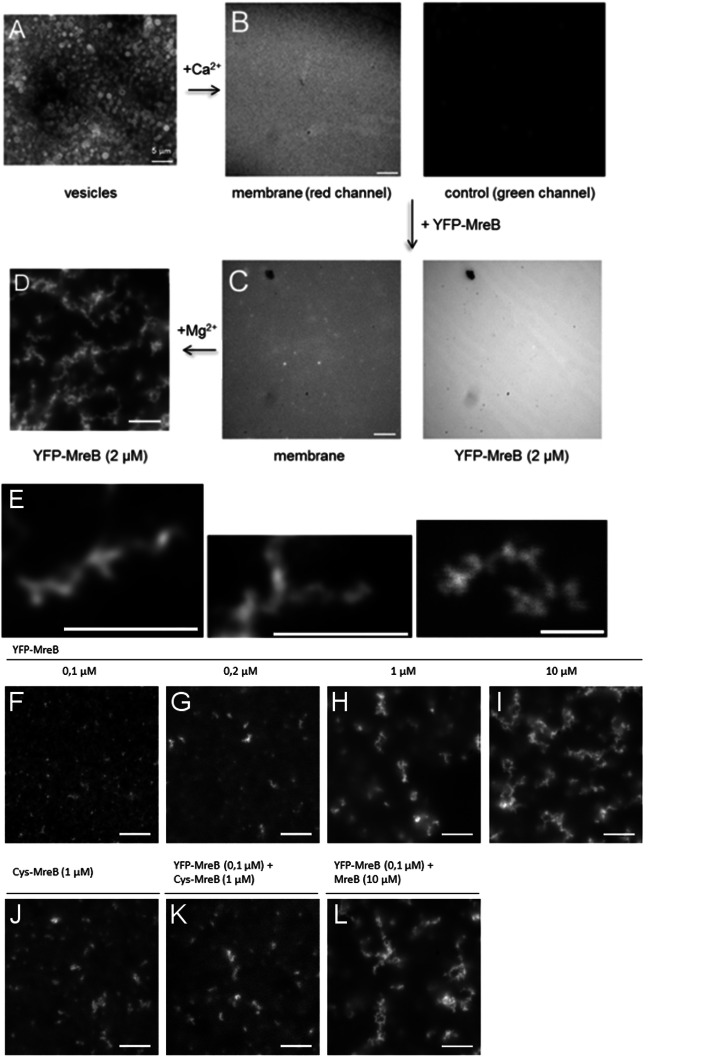
Assembly of YFP-MreB at a planar membrane in vitro. **a** Preparation of membrane vesicles, stained with FM4–64 (red fluorescent dye), **b** Calcium-induced fusion of vesicles establishes a flat membrane on top of a glass slide. Membrane stain in red, green channel shows background fluorescence in the YFP channel. **c** Diffuse localization of monomeric YFP-MreB on the membrane after washing with buffer. Note the small hole in the membrane in the upper left corner. **d** Addition of magnesium (10 mM) induces the formation of YFP-MreB filaments (2 μM) at the membrane. **e** STED images of individual YFP-MreB filaments (2 μM, 10 mM MgCl_2_, no KCl), and examples of branching and fusion of filaments. **f-i** Different concentrations of YFP-MreB as indicated (10 mM MgCl_2_, no KCl). **j** In vitro fluorescently labeled MreB carrying an additional cysteine residue integrated into the N-terminus of the protein (Cys-MreB) (2 μM, 10 mM MgCl_2_), (**k**) low concentration of YFP-MreB mixed with Cys-MreB, (**l**) Mixture of a low concentration of YFP-MreB and a high concentration of non-fluorescence-tagged MreB. Note that all constructs carry a Strep-tag for purification. White bars 2 μm

We next moved to imaging of YFP-MreB by fluorescence microscopy (FM). MreB has been shown to form polymers on various surfaces, including mica [10, 48, 49] and membranes [11], to which MreB from *B. subtilis* and from *E. coli* have intrinsic affinity via an internal hydrophobic loop or via an amphipathic N-terminal helix respectively [34, 38]. MreB filaments from *C. crescentus* have been visualized on their natural interaction surface, where they form flat sheet-like structures, with double filaments being the smallest visible form of individual filaments [11]. Visualization of MreB from a Gram positive bacterium on membranes has been missing so far. We therefore adapted a planar lipid bilayer system devised by the Schwille group [50], such that the formation of MreB filaments can be visualized on a biological membrane. Addition of calcium chroride (2 mM) to vesicles composed of lipids from *E. coli* cells (Fig. 3a) led to the formation of a planar membrane (Fig. 3b, c). The membrane was fluid as verified by FRAP analysis (suppl. Fig. S2A). The planar membrane could be stained with e.g. FM4–64, yielding homogeneous red fluorescence, and no fluorescence in the yellow channel (Fig. 3b). When monomeric YFP-MreB in low-salt storage buffer was added to the membrane, and the solution was subsequently washed with several volumes of storage buffer lacking YFP-MreB, a homogeneous staining of the membrane was observed (Fig. 3c). These experiments show that even non-polymerized YFP-MreB has membrane affinity, which strongly increases the local concentration of the protein at the cell membrane, and will enhance the efficiency of polymerization. In this case, 2D diffusion could be employed to find binding places at existing filaments, or to form nucleation centers. Addition of 10 mM magnesium chloride to purified YFP-MreB (2 μM) induced the formation of a network of filaments that were attached to the membrane, and remained attached even after washing of the reaction chamber (Fig. 3d). Occasionally, the planar membrane contained holes, at which filaments were never observed (suppl. Fig. S2b), revealing that MreB forms membrane-associated polymers in our experimental system. Induction of MreB filament formation led to a depletion of the homogeneous fluorescence at the membrane (Fig. 3d), indicating that membrane-attached MreB is efficiently incorporated into the filaments.

Using STED microscopy, we could visualize YFP-MreB filaments with a resolution of below 80 nm. Figure 3e reveals that filaments were not straight, but curved or even helical. Figure 3e shows that filaments could split up or merge, or twist, similar to what was observed using electron microscopy. Therefore, YFP-MreB filaments observed by FM appear to match the structures seen by EM.

The extent of filament formation was dependent on MreB concentration. At or below 0.1 μM YFP-MreB, we did not observe any elongated filamentous structures; instead, only focal structures were observed (Fig. 3f). These were distinct from the uniform distribution of MreB in the absence of magnesium ions (Fig. 3c), so it is likely that the fluorescent foci correspond to small MreB assemblies that could serve as nucleation centers. A minimal concentration of 0.2 μM was required to generate visible filaments (Fig. 3g), whose number and length increased with rising concentration; at 1 μM YFP-MreB, filaments of a length of up to 6.3 μm could be detected at the membrane (Fig. 3h, suppl. Fig. S3c). However, for many filaments, their ends were no longer detectable at the plane of the membrane, but extended away from the membrane. This became more pronounced with higher protein concentrations (Fig. 3i). Above 2 μM, filamentous YFP-MreB structures were mostly present in form of a branched network, where filaments split up and/or merged with other bundles or sheets of filaments. Even though the filamentous structures extended away from the membrane they were still bound to it at the base of the structure: when we washed the reaction chamber with different buffers, the filamentous structures stayed attached to the membrane. This enabled us to change conditions during later experiments. As the intracellular concentration of MreB has been determined to be around 5 μM [13], our data suggest that intracellular conditions must exist, or regulators, which prevent the formation of split up or merged filaments as seen in vitro.

We employed two strategies to investigate if the observed filaments are an artifact of the fluorescent protein fusion. Firstly, we added purified MreB to a concentration of YFP-MreB, which by itself does not lead to the formation of extended filaments. This approach also allows us to investigate if the architecture of filaments is altered when most of the structures were made up of non-tagged MreB. Addition of 10 μM MreB to 0.1 μM YFP-MreB and induction via magnesium or calcium ions resulted in the formation of highly extended filaments (Fig. 3l) that were visibly indistinguishable from those formed by 10 μM YFP-MreB by itself (Fig. 3j). Secondly, we incorporated a cysteine residue between the Strep-tag and the N-terminal residue of MreB; the N-terminus can be modified to carry a GFP and still be a functional fusion protein in vivo [29, 31]. Purified Cys-MreB was labelled with a fluorescein chromophore, and was added to the membrane, which led to the generation of filamentous structures after addition of magnesium ions (Fig. 3j); these structures had similar dimensions as YFP-MreB filaments. Additionally, when 0.1 μM YFP-MreB was mixed with non-stained Cys-MreB at 1 μM concentration, visible filaments arose that would not be seen at such a low concentration of YFP-MreB itself (Fig. 3k), suggesting that the stain does not influence the architecture of the filament sheets significantly. These experiments show that purified MreB can form extended filamentous structures on a flat membrane system in vitro*,* whose architecture is adequately reflected by YFP-MreB.

### Filamentous MreB structures have an average width of 90 nm and frequently exhibit a curved architecture

We took advantage of the fact that at or below 1 μM concentration of MreB, filaments formed a less extensive network, and attempted to gain information on their length, with the caveat that we had to use the length of extension away from the artificial cell membrane as an approximation for filament length. At 0.2 μM concentration, filaments could reach up to 2.3 μm, and had an average length of 1 μm, which increased to 1.5 μm at 0.5 μM, and to 1.85 μm at 1 μM YFP-MreB (Fig. S3A-C). At the latter concentration, filaments could extend to over 6 μm. Note that as will be explained below, monovalent ions strongly reduce filament size, and the above experiments were performed without the addition of K^+^. Nevertheless, they show that at given protein concentrations, MreB appears to polymerize into filaments whose length has a Gaussian rather than an arbitrary distribution.

**Fig. 4:**
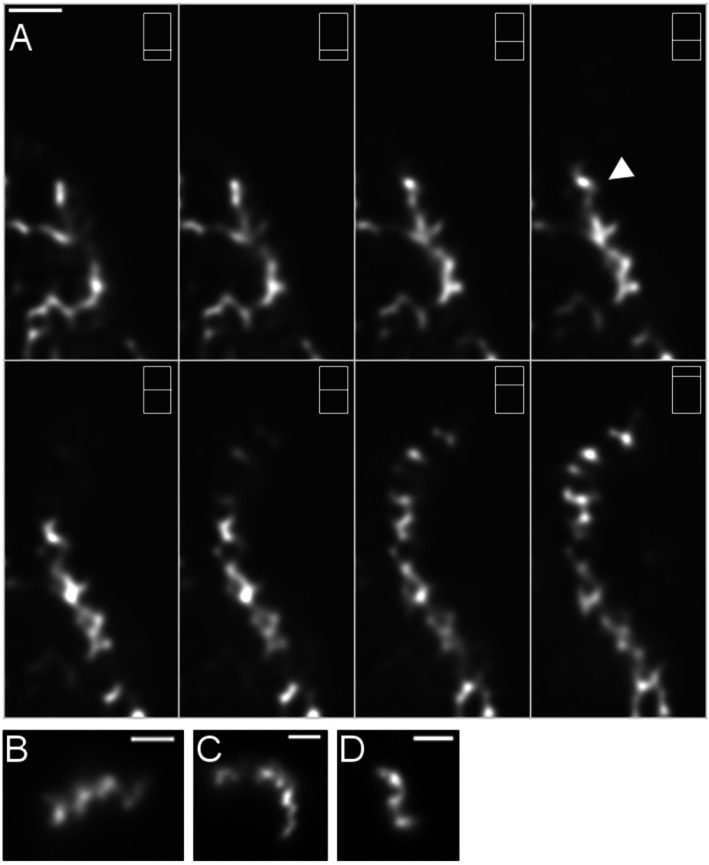
Formation of curved/helical YFP-MreB filaments extending from the planar membrane layer. **a** Z-stack through several planes away from the planar membrane, showing extension and branching of filaments. Apparent helical architecture is indicated by white triangles, different planes are indicated by white bar within rectangle. **b-d** Deconvoluted images of Z-stacks, B) single YFP-MreB filament, (**c**) single CFP-Mbl filament, (**d**) YFP-MreB filament in the presence of 100 mM potassium. Filament formation was induced through addition of 10 mM magnesium. White bars 1 μm

When Z-stacks of membrane-polymerized MreB were captured, the filaments had the appearance of helical structures (Fig. 4a, movies S1, S2 and S3). Similar to MreB (Fig. 4b), CFP-Mbl filaments frequently showed curved and helical appearance (Fig. 4c). Extended and curved filaments were also observed under high potassium (100 mM) concentrations (Fig. 4d).

We measured the width of YFP-MreB filaments, using STED microscopy. For smaller filaments (< 1 μm), the width was 91.7 ± 35 nm (*n* = 100), suggesting that the structures contain many MreB protofilaments, because the width of an individual MreB double protofilament would be about 8 to 9 nm. For larger filaments, the average width was determined to be 178 ± 95 nm (*n* = 100). In vivo, filament width was determined as 75 nm [29]. Our data have to be viewed with caution, keeping in mind that the observed networks of filaments are visibly quite dissimilar from structures formed in vivo.

### Divalent cations promote filament formation, while monovalent ion have an adverse effect

**Fig. 5:**
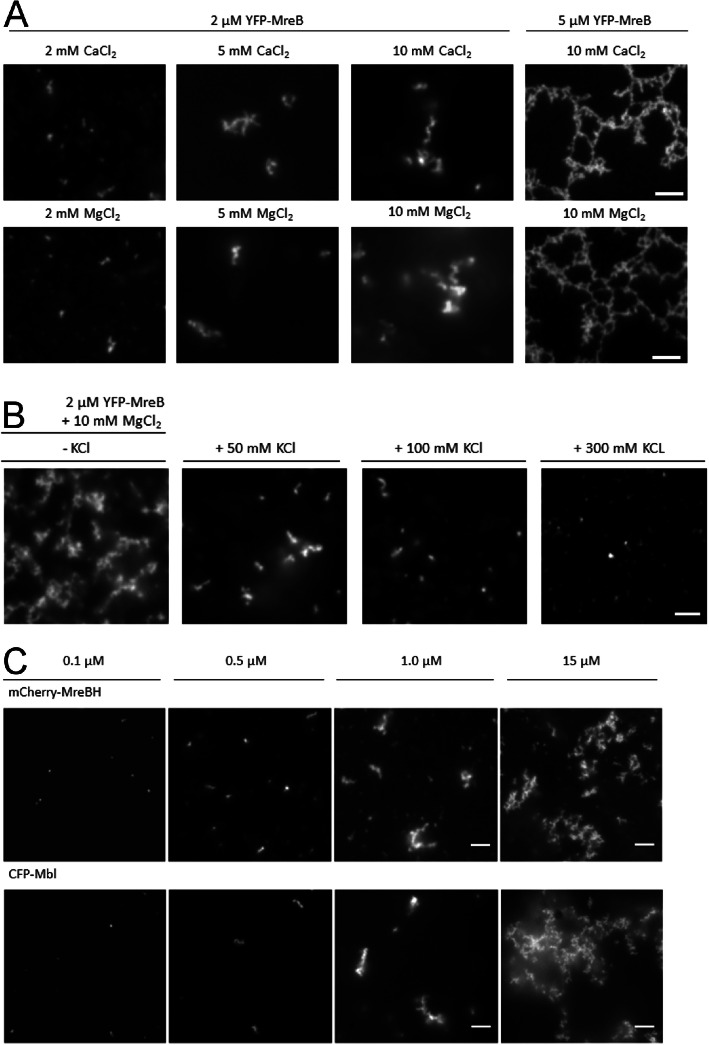
Fluorescence microscopy showing the dependency of filament formation of MreB paralogs on protein concentration, on a planar membrane. **a** Different concentrations of isolated YFP-MreB monomers after addition of different concentrations MgCl_2_, or of CaCl_2_, as stated above and below the panels, (**b**) YFP-MreB (2 μM) after addition of 10 mM MgCl_2_, in the presence of different concentrations of KCl as stated above the panels, (**c**) Different concentrations of CFP-Mbl or of mCherry-MreBH (after strep-purification) as stated above the panels after addition of 10 mM MgCl_2_, white bars 2 μm

MreB filament formation has been shown to be affected by ion concentrations in sedimentation and light scattering experiments [39, 40], which have been widely used to analyze polymerization of actin and actin-like proteins [51, 52]. Addition of increasing concentrations of magnesium chloride to purified MreB resulted in rapid increase in light scattering (Fig. S4A), indicative of rapid polymerization into filaments. Addition of potassium to the reaction strongly decreased the amount of scattering (Fig. S4B). We tested different ions to visualize the effect on the polymerization of MreB, employing 2 μM monomeric YFP-MreB in low-salt polymerization buffer. The addition of different concentrations of magnesium or of calcium ions visibly affected the formation of YFP-MreB filaments nucleating at the membrane (Fig. 5a). The amount of filaments increased in a magnesium ion dose-dependent manner, with visible saturation occurring at 10 mM magnesium (Fig. 5a). Additionally, the amount of protein used had a pronounced effect on the filament architecture (Fig. 5a). Using 10 mM MgCl_2_ and 5 μM YFP-MreB resulted in a level of filament formation that led to extended networks, similar to that of 10 mM CaCl_2_ and 5 μM YFP-MreB (Fig. 5a), showing that MreB filaments respond to both divalent ions in a similar fashion. Addition of CaCl_2_ showed visibly indistinguishable degrees of filament formation (Fig. 5a) compared with MgCl_2_. Therefore, membrane-associated MreB reacts to both divalent ions in a similar manner as MreB in solution.

Monovalent ions have been described to have a negative effect on the polymerization of MreB [40]. We used 10 mM MgCl_2_ to induce filament formation of MreB, in a solution containing different amounts of KCl. Employing the membrane system, we also observed an inhibitory effect of potassium on filament formation. Considerable inhibition was seen starting at concentrations of 50 mM KCl (Fig. 5b), and at 100 mM concentration, only short MreB filaments were visible by light microscopy (Fig. 5b). At 300 mM KCl, mostly foci and few filaments of YFP-MreB were detectable, indicating that only small assemblies of MreB exist at this concentration, but no longer extended filaments (Fig. 5b, see Fig. S5 for a quantitative analysis). Importantly, starting at 50 mM K^+^ concentration, no more split up filaments were observed (Fig. 5b), revealing that potassium counteracts non-productive filament interactions of MreB. The effect of 50, 100 or 300 mM NaCl was very similar to that of KCl (Fig. S6A). To test if chloride ions per se have an inhibitory effect on MreB when present at high concentrations, we added up to 300 mM magnesium chloride to the polymerization buffer and found that the formation of filaments was retained (Fig. S6B), ruling out that chloride plays a negative role. Thus, monovalent ions were effective in their inhibitory activity at roughly 10 fold higher concentrations than divalent ions. In the cell, the higher concentration of potassium compared to magnesium and calcium ions will therefore reduce the length of MreB filaments, and counteract filament splitting and thus branched meshwork formation.

We also tested if monovalent cations can induce the dissociation of preformed MreB filaments. The addition of 50, 100 or of 300 mM KCl did not show any effect on preformed YFP-MreB filaments (data not shown), revealing that once formed, monovalent cations no longer show an effect of MreB filaments, possibly, because their putative specific binding sites are now buried within the subunit interaction surface.

### MreB, Mbl and MreBH form mixed polymers that can laterally associate to preexisting filaments

We wished to gain further insight into the architecture of MreB filaments and to study the relation of the three MreB paralogs in vitro.

**Fig. 6:**
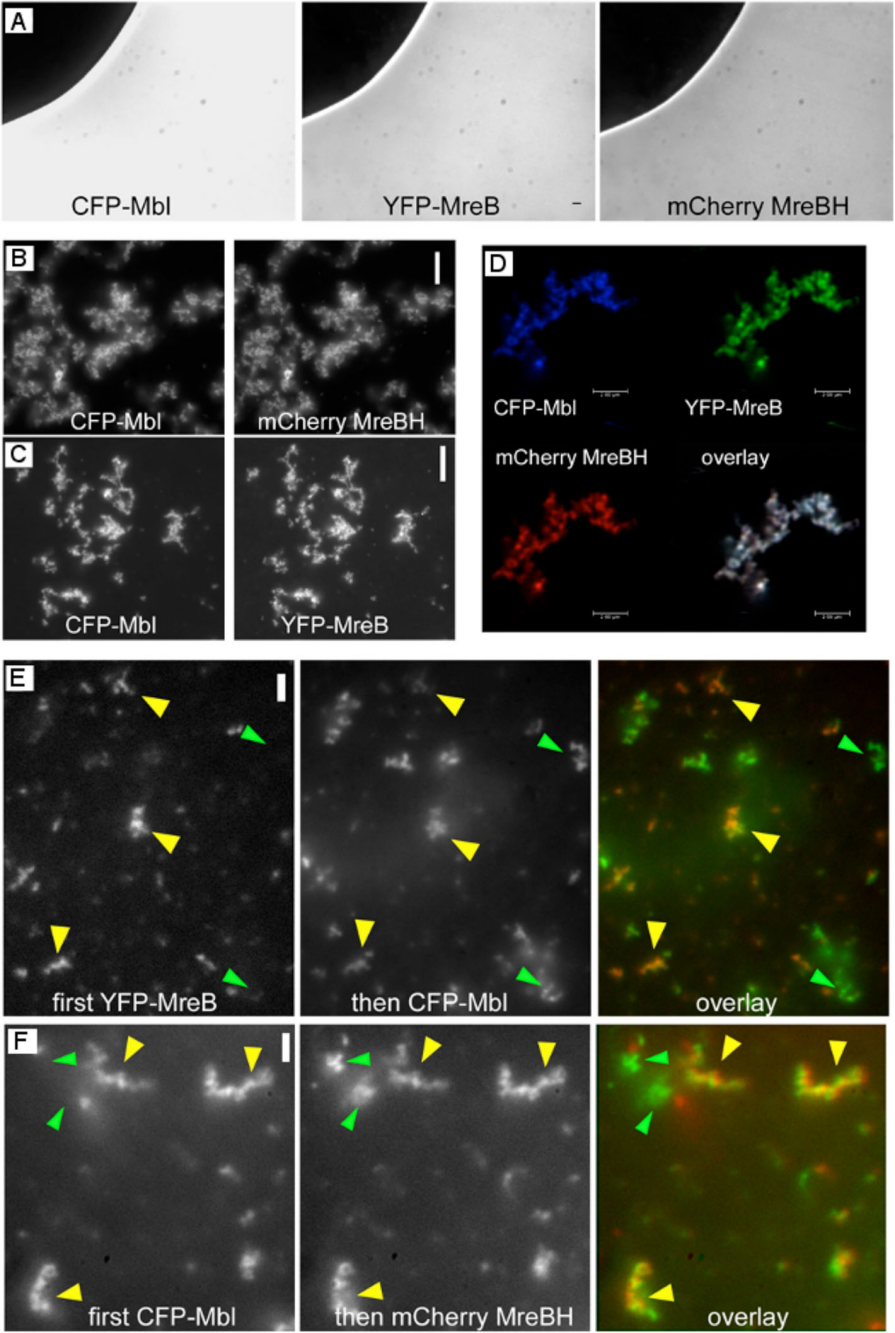
Co-localization of MreB paralogs in vitro. **a** MreB paralogs prior to induction of polymerization on a planar membrane. Note that there is no spectral bleed through between the panels, and that there is an area lacking membrane coating that fluctuates between the acquisitions. **b** Co-polymerization of CFP-Mbl and mCherry-MreBH, each 5 μM, (**c**) Co-polymerization of CFP-Mbl and YFP-MreB, both 2 μM, (**d**) Co-polymerization of all three MreB paralogs, each 1 μM, using confocal microscopy, (**e**) Addition of 2 μM CFP-Mbl (green in overlay) to pre-polymerized YFP-MreB (2 μM, red in overlay), yellow triangles indicate common filamentous structures, green triangles CFP-Mbl structures that assembled independent of preexisting YFP-MreB filaments. **f** Addition of 2 μM mCherry-MreBH (green in overlay) to preassembled CFP-Mbl (2 μM, red in overlay), yellow triangles in overlay indicate mCherry-MreB filaments assembled at preexisting YFP-MreB filaments, green triangles independent structures. Scalebars 2 μm

All three protein fusions exhibited affinity to the planar membrane (Fig. 6a). After inducing polymerization with 10 mM MgCl_2_, CFP-Mbl and mCherry-MreBH formed visible nucleation foci at 0.1 μM, and visible filamentous structures at 0.5 μM, whose length and number on the surface area increased with increasing protein concentration (Fig. 5c, Fig. S7). At 15 μM concentration, mCherry-MreBH formed filaments to a lesser degree than YFP-MreB or CFP-Mbl (Fig. 5c), but in general, all three MreB paralogs behaved very similarly with regard to polymerization on the membrane.

To assay any direct interaction between the paralogs, we mixed 1 μM of each, YFP-MreB, CFP-Mbl and mCherry-MreBH, which individually form only short filaments (Fig. 3h, Fig. 5c). When mixed, the joint formation of ion-induced, extended filaments was observed (Fig. 6d), showing that MreB paralogs cooperatively form single polymeric structures, in an additive manner. YFP-MreB also formed co-polymers with CFP-Mbl or mCherry-MreBH alone (Fig. 6c and data not shown), and likewise did CFP-Mbl and mCherry-MreBH (Fig. 6b).

The 100% overlay exemplified in Fig. 6d suggests that of all three proteins are closely associated within the joint molecules, when present at the initial stage of polymerization. These findings raise the interesting questions if MreB paralogs can associate with a preformed polymer, and if mixed polymers form by the addition of monomers to the ends of preexisting filaments, or in a lateral manner, or both. In the first case, we would expect that preformed filaments of one colour would contain extensions of another colour, in the latter case, preexisting filaments would be labeled with a second colour along their entire length. Figure 6e shows that CFP-Mbl was able to assemble at sites of previously formed YFP-MreB filaments, in addition to its independent filament formation. Likewise, mCherry-MreBH formed filaments along the length of pre-existing YFP-MreB filaments (data not shown), and also at preexisting Mbl filaments (Fig. 6f), while YFP-MreB could also attach to preformed CFP-Mbl filaments (data not shown). Of note, all co-polymers had an identical appearance, but we did not observe that an end of a co-filament had only a single colour (i.e. that of the lastly added paralog). Lateral association can be seen from a Z-stack shown in movie S4, in which 100 nm steps are imaged starting at and going away from the membrane. It can be seen that CFP-Mbl filaments shown in green are present in many focal planes together with MreB (in red), and extend further than many MreB structures. Lateral association is apparent from the many yellow filaments arising from a parallel existence of both MreB paralogs. These data show that filaments formed by one MreB paralog can be laterally extended by a second and third paralog, but are not extended at the end to a detectable degree, supporting the formation of lateral sheets of filaments.

### MreB forms filaments form between multilayered vesicles

**Fig. 7:**
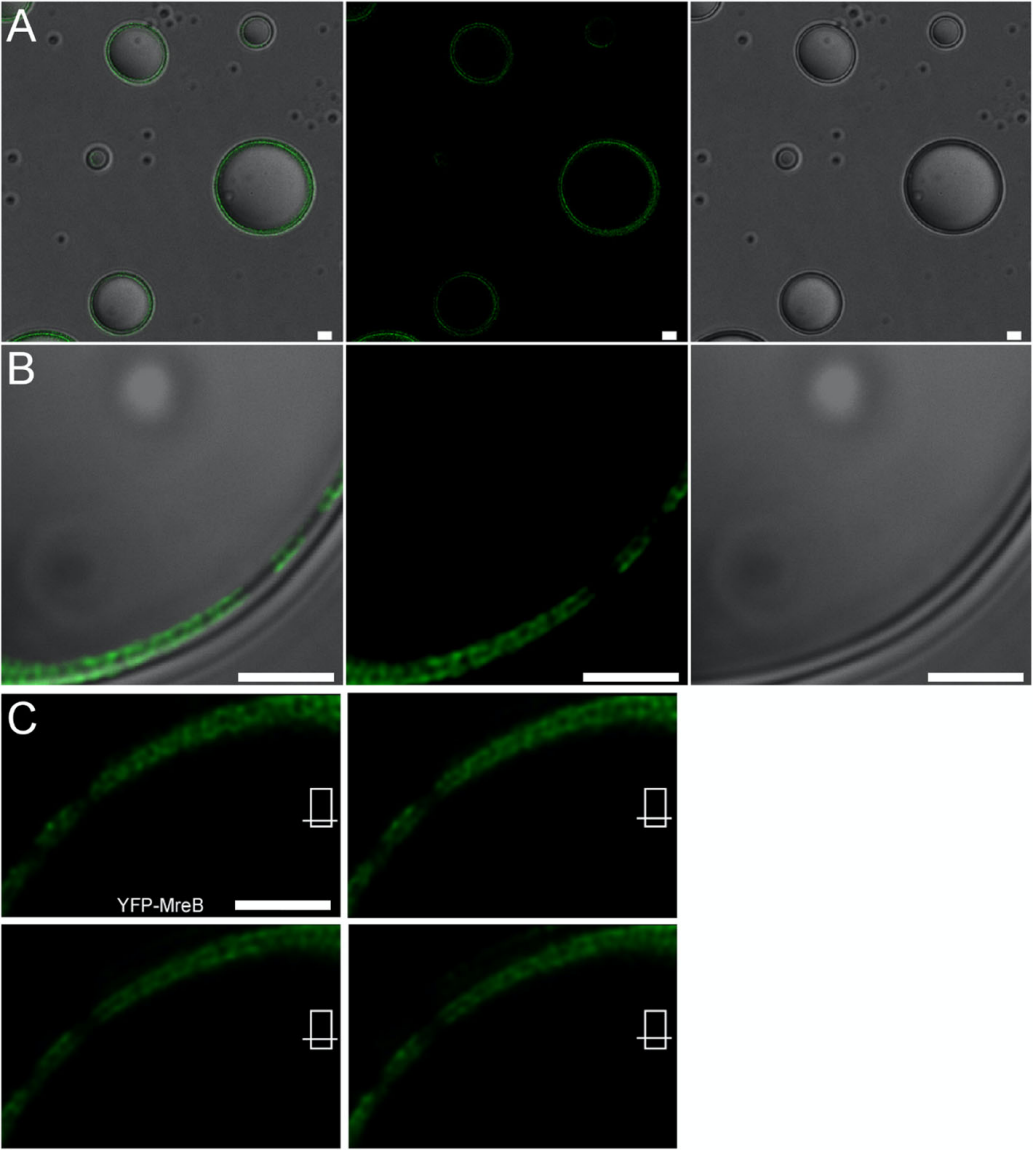
Polymerization of YFP-MreB within multilayered vesicles. **a** larger field of vesicles, (**b**) zoom into a multi layered vesicle, panels according to A): First panel overlay of fluorescence and DIC, second panel YFP fluorescence, third panel Nomarski DIC. **c** Z-stack zoom in into fluorescence channel of B). White bars 2 μm

MreB filaments can also form in solution, i.e. independent of a supporting bilayer. We wished to investigate if the proximity of a membrane favors the formation of filaments over that of in solution. We therefore generated lipid vesicles in the presence of non-polymerized YFP-MreB, such that frequently, multilayered vesicles would form. YFP-MreB was added to a planar membrane within a reaction chamber, additional lipid vesicles were added, and the mixture was removed from the chamber, vortexed and imaged after addition of calcium. This way, added vesicles (lacking YFP-MreB) were encircled by larger vesicles derived from the planar membrane and by non-polymerized YFP-MreB, which was then induced to form filaments. We observed the formation of YFP-MreB filaments at vesicle interfaces (Fig. 7a), especially where two vesicles were tightly packed (Fig. 7b). Interestingly, YFP-MreB filaments extended between the two juxtaposed biological membranes, and bound to both membranes, in an apparently helical pattern (Fig. 7c). These experiments show that the presence of a membrane, and especially two neighboring membranes favours the formation of filaments that are curved, similarly as observed on a planar membrane system, where filaments will extend away from the membrane.

## Discussion

Our work provides a visualization of filament formation for *B. subtilis* MreB and its paralogs Mbl and MreBH at biological membranes in vitro. It has been shown that MreB filaments have intrinsic membrane affinity [11, 34, 38], which in *E. coli* MreB is mediated by an N-terminal α-helix (wherefore an N-terminal YFP fusion to *E. coli* is non-functional [53]), and in *B. subtilis* MreB through an internal amphipathic helix, thus polymerization of MreB in the context of a biological membrane was an important goal. After having overcome problems with obtaining MreB in a non-polymerized, monomeric form, we induced polymerization of monomeric *B. subtilis* MreB and Mbl and MreBH on a planar lipid bilayer derived from the *E. coli* cell membrane, and visualized filamentous structures formed using electron microscopy as well as super-resolution fluorescence microscopy. The first important finding is that even non-polymerized MreB accumulates at a biological membrane, and therefore, monomers will also be enriched at the membrane in the cells, and not only polymeric MreB. This could increase the efficiency of filament formation, because MreB monomers can find each other or add to existing MreB filaments through 2D rather than 3D diffusion, possibly increasing the productivity of interaction with filaments. We also show that MreB filament sheets or bundles can add laterally to existing networks of filaments. At a low concentration of MreB (compared with actin), and upon a certain threshold concentration of magnesium or calcium ions, short filamentous structures were observed at the membrane, indicating that MreB filaments can efficiently nucleate at the cell membrane and rapidly form micrometer-long bundles or sheets of filaments dependent on the presence of divalent ions, and in the absence of additional nucleotides (note that nucleotides could remain bound to MreB during purification). However, at higher protein concentrations (above 1 μM, while about 5 μM MreB was calculated to be present in *B. subtilis* cells [13]), MreB filaments started to split up and/or merge with other filaments, such that a network of filaments arose. Thus, at physiological protein concentrations and at low concentrations of monovalent ions, MreB formed branched networks of filaments, apparently through interactions between different sheets or bundles of filaments. These structures appear to be an irregularity, because in vivo, MreB filaments are relatively uniform and rarely branched [29, 30]. Of note, because the ratio of volume to membrane surface in the reaction chamber used in our studies is lower than that in the rod shaped bacterial cell, and as MreB has a preference for membrane-attachment even as a monomer (or at least being in the non-polymerized state), conditions used in our in vitro work and those existing in the cell may vary considerably.

At and above 50 mM K^+^ concentrations, MreB only formed shorter, less branching structures. Therefore, intracellular potassium levels are able to counteract non-productive filament interactions of MreB. *B. subtilis* cells have a basal potassium pool of 180 to 300 mM during growth, and 200 mM during exponential phase [54, 55]. It is not yet clear how much of this pool is free or tightly bound. In any event, potassium ion concentrations in vivo are high enough to effectively reduce the length of MreB filaments as judged from our in vitro analyses, because this effect was already seen at concentrations of 50 mM K^+^ or higher. However, in vitro, short MreB filaments were seen even at 250 mM K^+^, so intracellular potassium levels will not fully prevent polymerization of MreB and likely play a regulatory role. There is no detectable free sodium in *B. subtilis* cells, so a negative effect of Na^+^ will be negligible. On the other hand, 1 mM free Mg^2+^ has been measured inside Gram negative bacteria [56], and may be higher in *B. subtilis*, because Gram positive bacteria have a higher internal turgor pressure than Gram negatives [57]. No data are available for intracellular calcium concentrations, which are expected to be rather low, within the same range as those of magnesium. Therefore, cellular concentrations of divalent ions are high enough to induce or contribute to the formation of several micron-long filaments in vitro. Taking into account that additional stabilizing and destabilizing factors will exist in cells, the intracellular ratio between monovalent and divalent ions will strongly affect average length of MreB filaments in vivo*,* alongside intracellular concentrations of MreB. Indeed, at low potassium and low MreB concentrations, where filaments were still attached to the membrane, we observed a Gaussian-like distribution of filament length, though measurement was difficult, given the curved and branched nature of the samples. Additionally, MreB interacts with translation elongation factor EF-Tu in vivo and in vitro [58, 59]. EF-Tu enhances the efficiency of filament formation of MreB in vitro [42], indicating that EF-Tu may act as stabilizing factor in vivo.

Similar to MreB, the extent of filament formation of Mbl and of MreBH was strongly affected by physiological ion concentrations, as well as by protein levels. Magnesium and calcium ions strongly favored filament formation, while potassium and sodium counteracted this process. A very similar behavior of all three MreB paralogs with respect to protein concentrations and magnesium/ calcium effects were observed. Once formed, MreB, Mbl and MreBH filaments were very stable and did not show shrinking or extension in vitro for at least 10 min. Recent experiments have suggested that the circumferential movement of MreB filaments in bacterial cells does not rely on treadmilling, but that entire MreB filaments move [30, 31]. Our data suggest that MreB filaments can rapidly form but then remain stable in their length on the scale of minutes or more. MreB filaments also remain stable in vivo, with observed internal remodeling [35, 42].

We further show that besides very similar polymerization properties in vitro*,* MreB paralogs MreB, Mbl and MreBH co-polymerize into a single filamentous structure. Paralogs can add laterally to existing filaments, in agreement with the variable width of the filament sheets seen in EM analysis. Thus, like MreB from Gram-negative bacteria, MreB from Gram-positives may form flat sheets of straight double- (antiparallel) protofilaments directly underneath the cell membrane. It will be interesting to obtain even higher resolution insight on *B. subtilis* MreB structures in vitro, and to investigate which cellular factors are required to mediate MreB filament formation as observed within *B. subtilis* cells; RodZ and EF-Tu are two candidates for this task.

## Methods

### Expression and purification of strep-tagged proteins

For heterologous protein expression in *E. coli* BL21 (λDE3), transformed *E. coli* cells harboring the respective plasmid were inoculated into 2 × 1 l of LB medium in 2 l chicaned flasks supplemented with streptomycin or ampicillin, respectively. The media was, if not otherwise stated, supplemented with 500 mM Sorbitol and 1 mM Betaine. Prior to induction of protein expression by addition of IPTG (1 mM), cells were grown at 37 °C until to an OD of 0.6–0.8 and subsequently grown at 18 °C ON. Cell pellets were disrupted via French press in appropriate buffer (100 mM Tris HCl, 300 mM NaCl, 1 mM EDTA, 0.2 mM ATP, 5% Glycerol pH 7.5) containing a mix of protease inhibitors (Complete, Roche). The lysate was cleared by centrifugation and the strep-tag fused proteins were purified by affinity chromatography using gravity flow columns with streptactin sephrose (IBA, Göttingen). Protein fusions were concentrated by ultrafiltration (concentrator columns, Vivaspin, Sartorius Stedim Biotech) and finally re-buffered in low salt polymerization buffer (5 mM TRIS-HCl, 0.1 mM CaCl_2_, 0.2 mM ATP, pH 7.5) by dialysis (dialysis chambers, Slide-A-Lyzer 7 K Dialysis Cassettes, Thermo Scientific) in order to obtain the monomeric forms via size-exclusion chromatography (Superdex 200 Increase 10/300 GL, GE Healthcare Life Sciences).

### Sucrose density gradient

For assessment of molecular weight of proteins, a 5–15% (w/v) sucrose density gradient was prepared with the appropriate buffer for the respective protein fractions, isolated from size exclusion gel chromatography. The gradient was prepared by filling an ultracentrifuge tube with 5% sucrose solution, then underlaying a 15% sucrose solution. The Gradient Station ip (BIOCOMP) was used to mix the gradient. After loading of the protein sample, the gradient was placed in an Optima XPN-80 (Beckmann Coulter) with a SW40Ti swing-rotor. Centrifugation was performed with 38.000 x g at 4 °C for approximately 16 h (the centrifuge was set to swing without deceleration). Gradients were afterwards fractioned from the top in 1 ml increments. For reference a gel filtration standard (Biorad) was used, containing a mixture of Vitamin B12 (1.35 kDa), Myoglobin (horse, 17 kDa), Ovalbumin (chicken, 44 kDa), Gamma-Globulin (bovine, 158 kDa) and Thyroglobulin (bovine, 670 kDa).

### Mass photometric analysis of protein stoichiometry

The Refeyn OneMP mass photometer was used to determine stoichiometry of protein isolates in solution. To calibrate the instrument, Native Mark protein standard (Biorad) was diluted 50 fold in sample buffer at room temperature. 2 μl of diluted calibration mixture was mixed with 18 μl of sample buffer on silicone wells on a cleaned microscope slide (170 ± 5 μm thickness, Marienfeld). We used the 66, 146, 480 and 1048 kDa peaks for a four-point calibration. For the measuements, 18 μl buffer were pre-loaded into a silicone well, then 2 μl of 500 nM protein solution was mixed in prior to acquisition, yielding a final concentration of 50 nM. We collected 6000 frames for each protein using default instrument parameters. Data was analyzed with the DiscoverMP software provided by Refeyn, using default parameters for event extraction and fitting. Frames affected by strong vibration or aggregates moving across the image were manually excluded.

### Construction of strep-Cys-MreB and labeling with thiol reactive dye

*Bacillus subtilis* MreB fused to strep-tag was qualified for labeling with the thiol-reactive dye BODIPY® FL maleimide (Molecular Probes) by insertion of one cysteine residue exposed at the surface of the protein fusion. To this end, site-directed PCR mutagenesis was performed with the primers CATCCGCAGTTTGAAAAATGCATGTTTGGAATTGGTGC (strep-MreB-C-up) and GCACCAATTCCAAACATGCATTTTTCAAACTGCGGATG (strep-MreB-C-dw) (additional cystein codon between strep-tag sequence and *mreB* sequence) and with pJS36 as DNA template generating pCR8. The expression product termed Strep-Cys-MreB was labeled with the dye according to the protocol of the manufacturer (BODIPY® FL maleimide, Molecular Probes). The labeling reaction was performed with 5 mM purified Strep-Cys-MreB in low salt buffer (5 mM TRIS-HCl, 0.1 mM CaCl_2_, 0.2 mM ATP, pH 7.5) and 50 mM dye at stirring conditions for 2 h at room temperature. The reaction was quenched with 10 mM β-mercaptoethanol before applying the protein fusion on supported lipid bilayers.

### Investigation of proteins on supported lipid bilayer

The polymerization reactions of purified *B. subtilis* MreB proteins on supported membranes were performed in reaction chambers consisting of the top of an Eppendorf tube (diameter 85 mm) glued on glass slides with UV adhesive glue (Norland Optical Adhesive 63, Norland Products, Cranbury, NJ), using a reaction volume of 200 μl. For the formation of supported membranes, the suspension of 4 mg/ml polar lipid extracts (Avanti Polar Lipids, Alabaster, AL) in ddH_2_O was sonicated generating small unilamellar vesicles (SUVs). Fusion of diluted SUVs (final concentration 0.8 mg/ml) to a homogeneous membrane in the reaction chamber was induced by the addition of CaCl_2_ to a final concentration of 2 mM in a 150 μl lipid solution. After 1 h of incubation at room temperature, the membrane was washed four times with 5 ml low salt buffer (5 mM TRIS-HCl, 0.1 mM CaCl_2_, 0.2 mM ATP, pH 7,5) to remove non-fused vesicles. Subsequently, purified protein(s) in the same low salt buffer was / were added. Incubation at room temperature for 15 min allowed the monomeric proteins to attach to the membrane. Polymerization of the proteins was induced by the addition of divalent cations (if not stated otherwise: 10 mM MgCl_2_), followed by 15 min incubation and subsequent washing of the chamber with several volumes of low-salt polymerization buffer, prior to microscopy.

### Fluorescence microscopy

For microscopy analyses, different concentrations and mixtures of purified protein-fusions, in low salt polymerization buffer solution (5 mM TRIS-HCl, 0.1 mM CaCl_2_, 0.2 mM ATP, pH 7.5), were applied to the membrane inside of a reaction chamber. Epifluorescence microscopy was performed using a Zeiss Axioobserver Z1 microscope (Carl Zeiss, Jena, Germany) with a 1.45 numerical aperture objective and a Photometrics Cascade EM-CCD camera (Photometrics, Tucson, AZ). The data was processed with MetaMorph 6.3 software (Meta Imaging Software, Molecular Devices, Sunnyvale, CA) and subsequent image analysis was performed using ImageJ (National Institutes of Health, Bethesda, MD). Superresolution microscopy was performed utilizing the STED (Leica) technique, using a Leica G-STED SP8 microscope. Images were captured with 400 Hz (three to four line scans).

### Electron microscopy

For transmission electron microscopy (TEM), the purified, monomeric proteins in low-salt polymerization buffer were directly applied to carbon-coated 400 mesh copper grids followed by the addition of 10 mM MgCl_2_ to induce polymerization of the appropriate samples. After blotting to filter paper, the samples were negatively stained with 2% uranyl acetate for 20 s and washed twice with double distilled water. Electron microscopy was carried out at 120 kV on a JEOL JEM-2100 transmission electron microscope (JEOL, Tokyo, Japan) equipped with a LaB_6_ cathode and a 2 k × 2 k fast scan CCD camera F214 and EMMenu4 (TVIPS, Gauting, Germany).

### Multilayered vesicle formation

A supported membrane was formed as previously described, to which non-fused small vesicles were added (1 mg/ml). The reaction chamber was then incubated at 37 °C for 1 h. Mixing / vortexing of the solution resulted in the occasional formation of multilayered vesicles of various sizes (MLVs). If monomeric MreB was added under polymerizing conditions, prior to the formation of MLVs, the protein was incorporated inside of the vesicles.

### Dynamic light-scattering

Light scattering was measured at 418 nm after excitation at 315 nm in a Shimadzu RF-5001PC or PerkinElmer LS55 fluorimeter. The scattered light intensity was measured at an angle of 90° from the direction of the incident light. The temperature was set in the cuvette (Quartz SUPRASIL Ultra-micro from PerkinElmer) at 25 °C. Appropriate concentration of proteins samples was added to the polymerization buffer to a final volume of 100 μl. The mixture was equilibrated at 25 °C for 2 min before adding magnesium chloride which triggered the polymerization.

## Supplementary information

**Additional file 1 Movie S1.** STED microscopy Z-stack (100 nm step size, deconvolved) of YFP-MreB on a flat membrane, 3 fps.

**Additional file 2 Movie S2.** Volume rendering from STED Z-stacks (100 nm step size) of YFP-MreB on a flat membrane.

**Additional file 3 Movie S3.** Confocal microscopy, Z-stack (100 nm step size) of YFP-MreB on a flat membrane, 3 fps.

**Additional file 4 Movie S4.** Confocal microscopy, Z-stack (100 nm step size) of YFP-MreB polymerized on a flat membrane, followed by washing and second polymerization of CFP-Mbl, 3 fps.

**Additional file 5: Figure S1.** Electron microscopy of negatively contrasted MreB filaments.

**Additional file 6: Figure S2.** Fluorescence recovery after photobleaching (FRAP) analysis of a lipid membrane used in the analysis. At time point “0 s”, an area of 1 μm is bleached (“FRAP”) and recovers in the following 4 s interval acquisitions. B) YFP-MreB (5 μM) polymerized on a planar membrane using 5 mM MgCl_2_. Occasionally, membranes are patchy and contain holes, where YFP-MreB filaments are not observed.

**Additional file 7: Figure S3.** Measurement of filament length of YFP-MreB nucleated at a planar membrane, from Z-stacks taken by epifluorescence microscopy. The concentrations of the proteins are stated above the panels, average filament length on the right (SD = standard deviation).

**Additional file 8: Figure S4.** Dynamic light scattering of purified MreB (5 μM, see Fig. 1). A) Scattering dependent on different concentrations of magnesium as indicated. B) Scattering in buffer containing 5 mM magnesium, dependent on different concentrations of potassium as indicated.

**Additional file 9: Figure S5.** Quantification of maximum projections of Z-stacks from YFP-MreB filaments at different ion concentrations. A): Mean of the total integrated fluorescence intensity for maximal projections of 10–15 micrograph stacks (512 × 512 pixel) for different ion conditions. Each condition contains 2 μM YFP-MreB supplemented with 10 mM MgCl_2_ and was treated as previously described. (BI-EI): Exemplary surface blots, giving three-dimensional graphs of the intensities of pixels in grayscale, for maximal projections of YFP-MreB fluorescence micrograph stacks with B): no KCl added; C): 50 mM KCl added; D): 100 mM KCl added; E): 300 mM KCl added. (BII-EII): Exemplary planes of YFP-MreB micrograph stacks at the indicated ion conditions. Scale bar 2 μm.

**Additional file 10: Figure S6.** Fluorescence microscopy showing the dependency of filament formation of MreB on sodium or magnesium chloride concentration on a planar membrane. A) 2 μM of purified monomeric YFP-MreB after addition of 10 mM MgCl_2_ in the presence of different concentrations of NaCl as stated above the panels. B) 2 μm YFP-MreB addition of different amounts of MgCl_2_ as stated above the panels. White bars 2 μm.

**Additional file 11: Figure S7.** Measurement of filament length of CFP-Mbl or of mCherry-MreBH nucleated at a planar membrane, from Z-stacks taken by epifluorescence microscopy. The concentrations of the proteins are stated above the panels, average filament length on the right (SD = standard deviation).

**Additional file 12.**

## Data Availability

The datasets generated and/or analysed during the current study are available in the “data_UMR/Forschungsdatenrepositorium repository”, 10.17192/fdr/33.

## Abbreviations

ATP: Adenosine triphosphate
CFP: Cyan fluorescent protein
EDTA: Ethylenediaminetetraacetic acid
GFP: Green fluorescent protein
STED: Stimulated emission depletion
YFP: Yellow fluorescent protein
