# Supplementary figures and images for: Polymerization of Bacillus subtilis MreB on a lipid membrane reveals lateral co-polymerization of MreB paralogs and strong effects of cations on filament formation
Source: BMC Mol Cell Biol. 2020 Nov 4;21:76. doi: 10.1186/s12860-020-00319-5 (PMC7641798; doi:10.1186/s12860-020-00319-5)

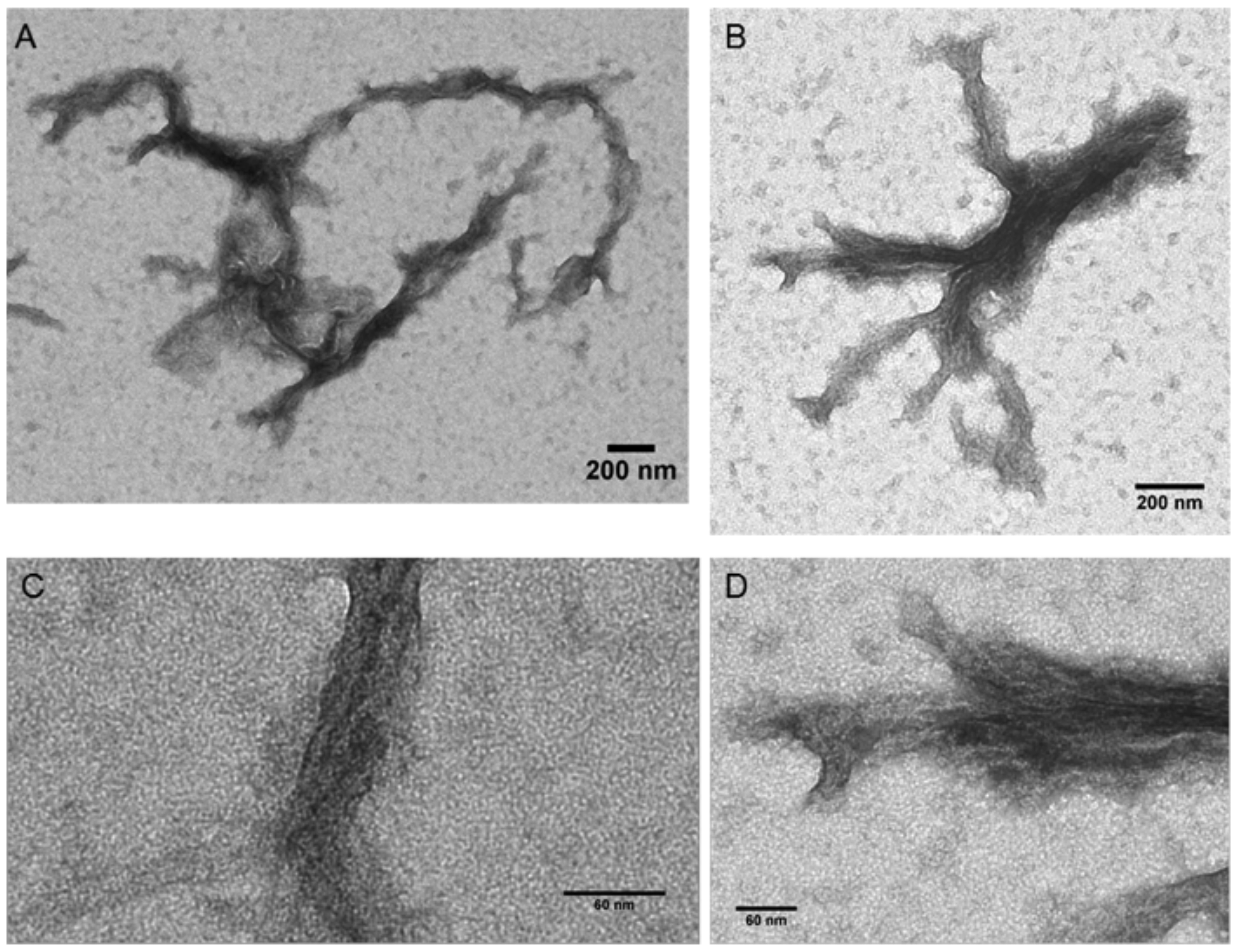

Supplement: Supplementary file 5 — Additional file 5: Figure S1. Electron microscopy of negatively contrasted MreB filaments. [file 12860_2020_319_MOESM5_ESM.tif]

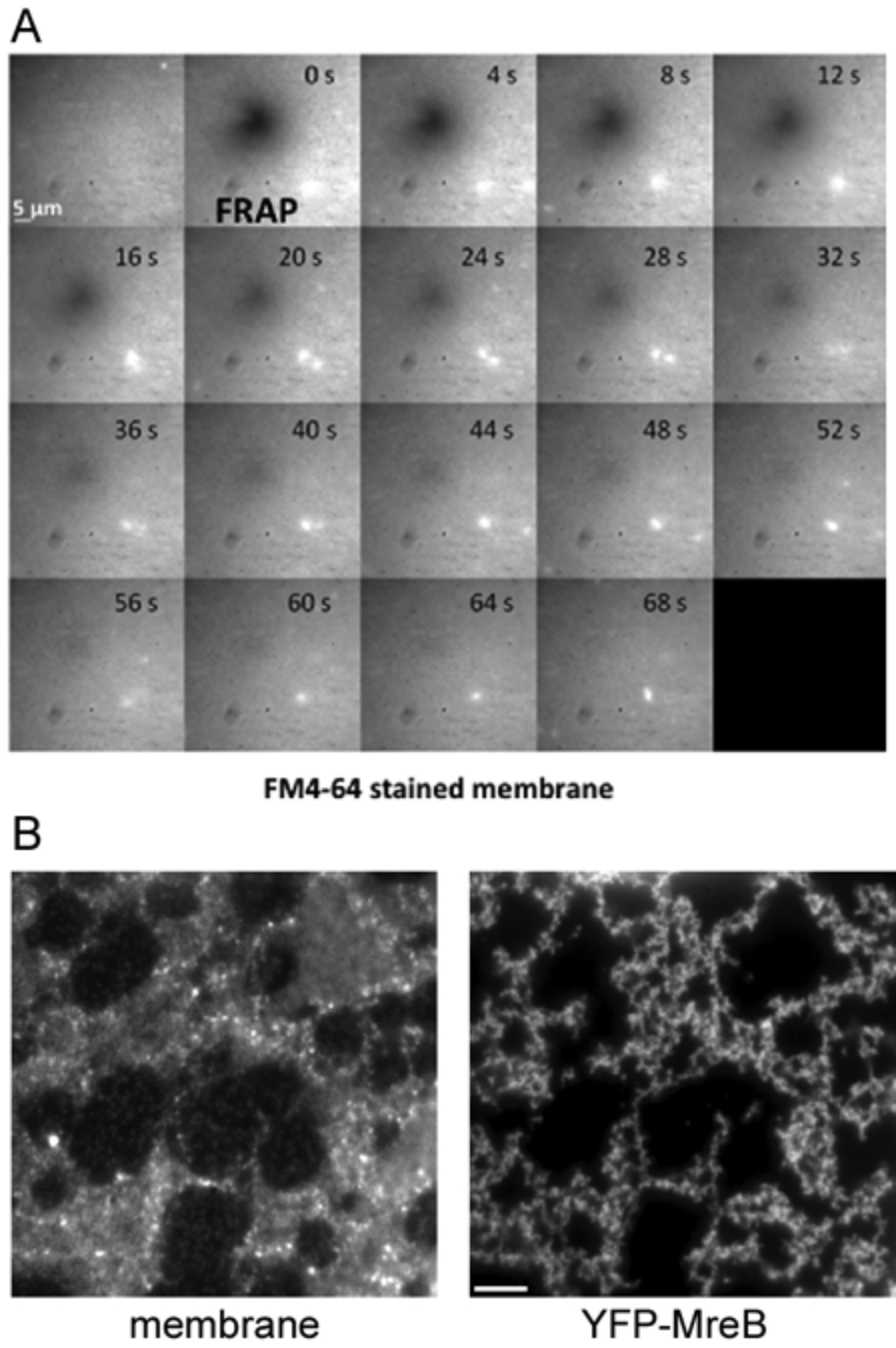

Supplement: Supplementary file 6 — Additional file 6: Figure S2. Fluorescence recovery after photobleaching (FRAP) analysis of a lipid membrane used in the analysis. At time point “0 s”, an area of 1 μm is bleached (“FRAP”) and recovers in the following 4 s interval acquisitions. B) YFP-MreB (5 μM) polymerized on a planar membrane using 5 mM MgCl2. Occasionally, membranes are patchy and contain holes, where YFP-MreB filaments are not observed. [file 12860_2020_319_MOESM6_ESM.tif]

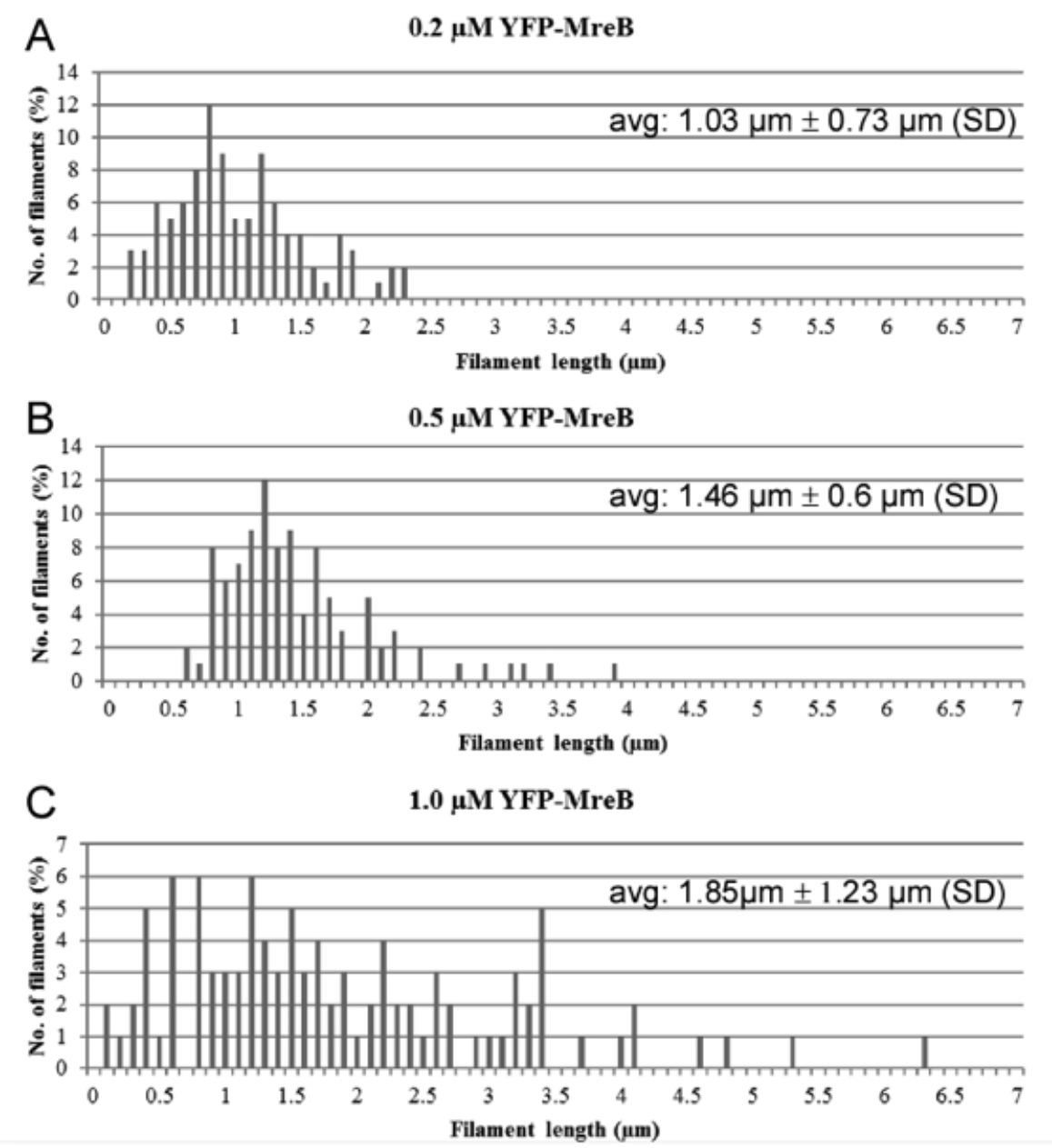

Supplement: Supplementary file 7 — Additional file 7: Figure S3. Measurement of filament length of YFP-MreB nucleated at a planar membrane, from Z-stacks taken by epifluorescence microscopy. The concentrations of the proteins are stated above the panels, average filament length on the right (SD = standard deviation). [file 12860_2020_319_MOESM7_ESM.tif]

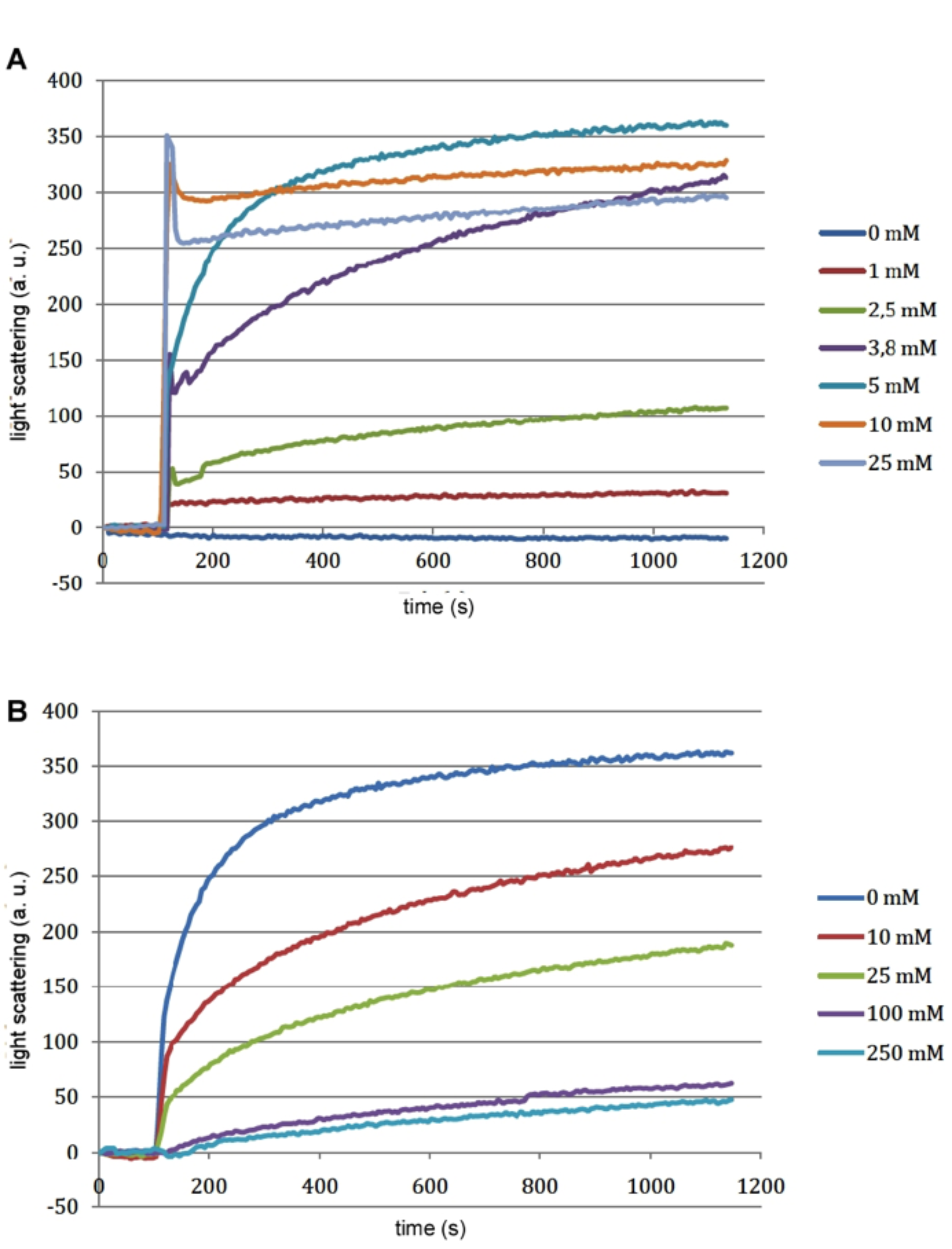

Supplement: Supplementary file 8 — Additional file 8: Figure S4. Dynamic light scattering of purified MreB (5 μM, see Fig. 1). A) Scattering dependent on different concentrations of magnesium as indicated. B) Scattering in buffer containing 5 mM magnesium, dependent on different concentrations of potassium as indicated. [file 12860_2020_319_MOESM8_ESM.tif]

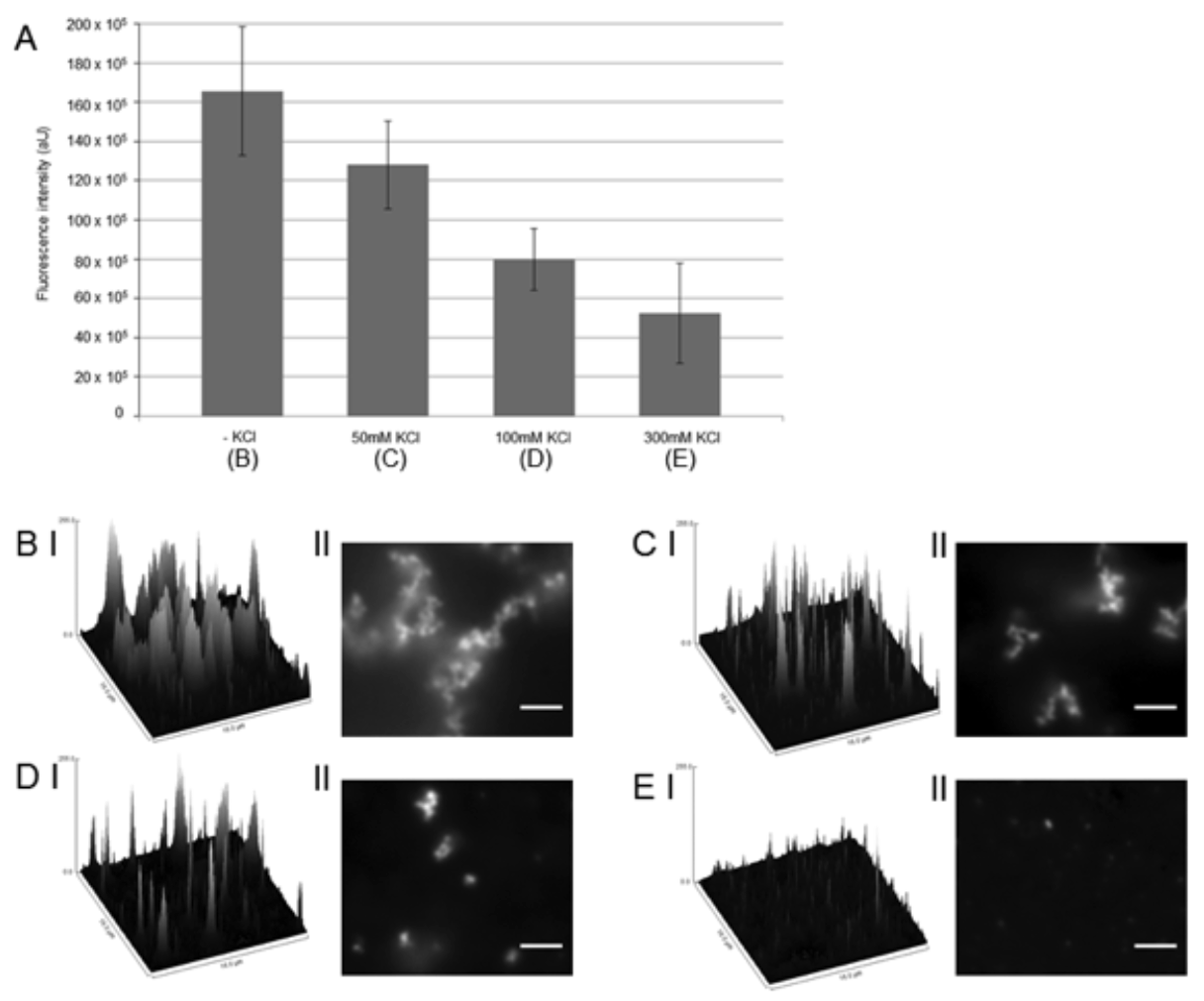

Supplement: Supplementary file 9 — Additional file 9: Figure S5. Quantification of maximum projections of Z-stacks from YFP-MreB filaments at different ion concentrations. A): Mean of the total integrated fluorescence intensity for maximal projections of 10–15 micrograph stacks (512 × 512 pixel) for different ion conditions. Each condition contains 2 μM YFP-MreB supplemented with 10 mM MgCl2 and was treated as previously described. (BI-EI): Exemplary surface blots, giving three-dimensional graphs of the intensities of pixels in grayscale, for maximal projections of YFP-MreB fluorescence micrograph stacks with B): no KCl added; C): 50 mM KCl added; D): 100 mM KCl added; E): 300 mM KCl added. (BII-EII): Exemplary planes of YFP-MreB micrograph stacks at the indicated ion conditions. Scale bar 2 μm. [file 12860_2020_319_MOESM9_ESM.tif]

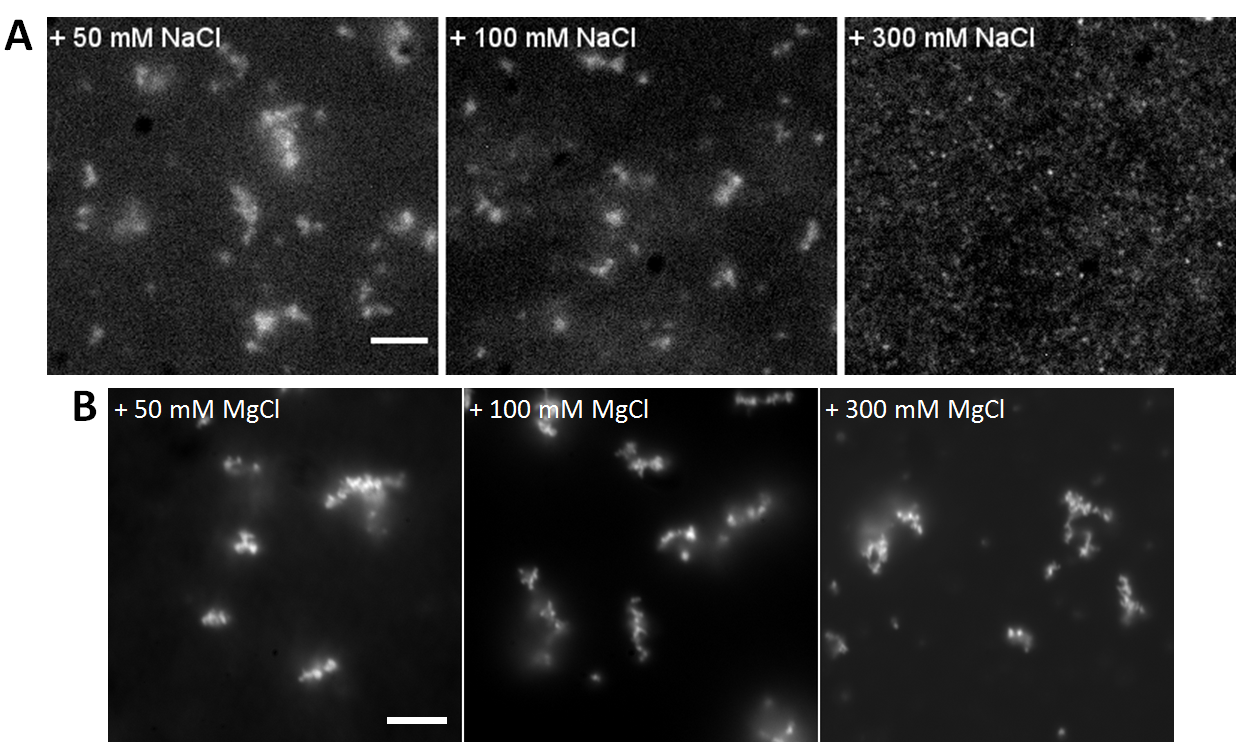

Supplement: Supplementary file 10 — Additional file 10: Figure S6. Fluorescence microscopy showing the dependency of filament formation of MreB on sodium or magnesium chloride concentration on a planar membrane. A) 2 μM of purified monomeric YFP-MreB after addition of 10 mM MgCl2 in the presence of different concentrations of NaCl as stated above the panels. B) 2 μm YFP-MreB addition of different amounts of MgCl2 as stated above the panels. White bars 2 μm. [file 12860_2020_319_MOESM10_ESM.tiff]

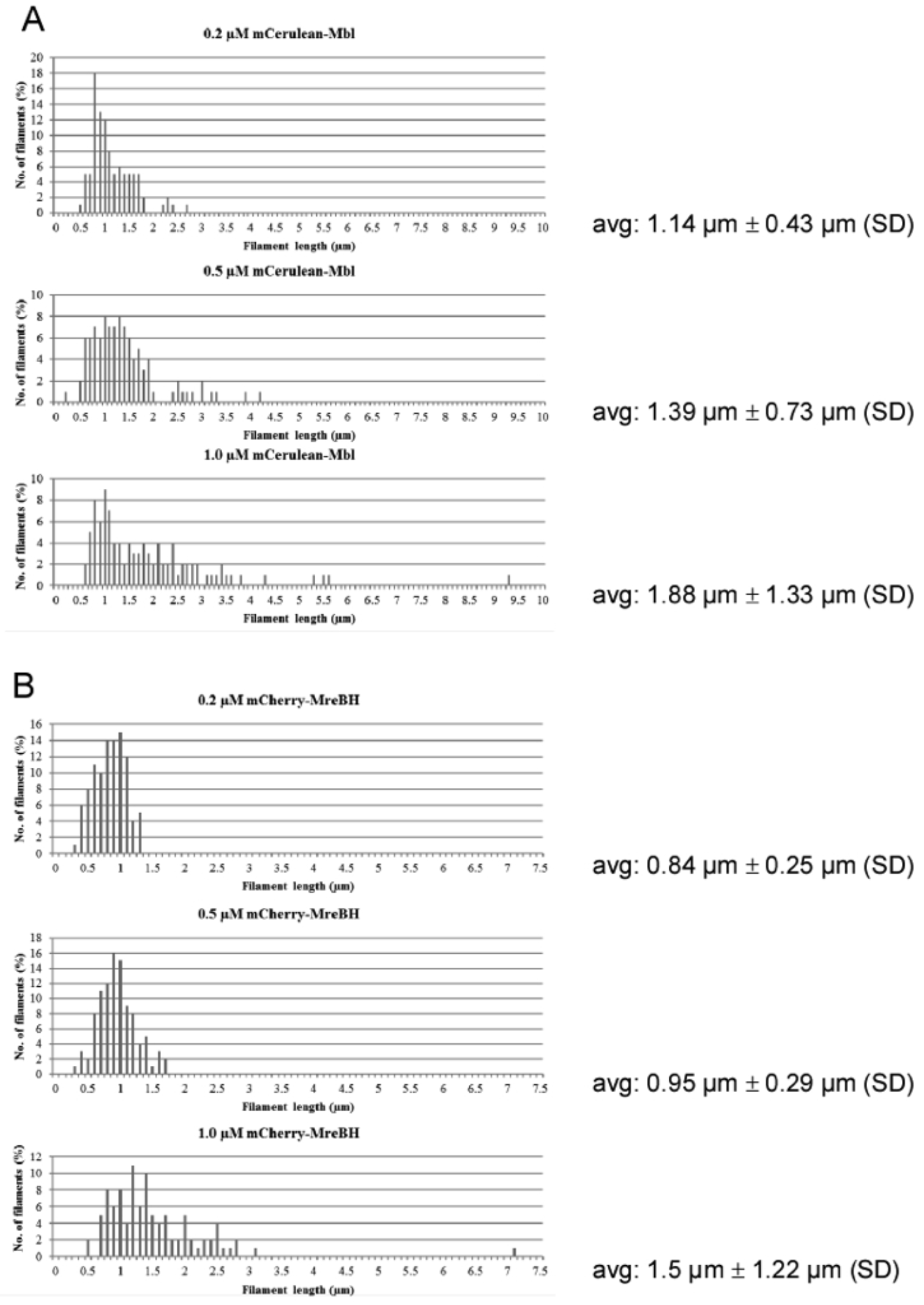

Supplement: Supplementary file 11 — Additional file 11: Figure S7. Measurement of filament length of CFP-Mbl or of mCherry-MreBH nucleated at a planar membrane, from Z-stacks taken by epifluorescence microscopy. The concentrations of the proteins are stated above the panels, average filament length on the right (SD = standard deviation). [file 12860_2020_319_MOESM11_ESM.tif]
