# Supplementary material for: Polymerization of Bacillus subtilis MreB on a lipid membrane reveals lateral co-polymerization of MreB paralogs and strong effects of cations on filament formation
Source: BMC Mol Cell Biol. 2020 Nov 4;21:76. doi: 10.1186/s12860-020-00319-5 (PMC7641798; doi:10.1186/s12860-020-00319-5)

Full length blots corresponding to figure 1:

Sucrose gradient (5 - 15 %) of isolated monomer peaks of YFP-MreB, CFP-Mbl, mCherry-MreBH. Biorad gel-filtration standard was used as a reference. (Marker proteins appearing in lane 1: Myoglobin, 2: Ovalbumin, 4: Gamma-Globulin, 10: Thyroglobulin; YFP-MreB, CFP-Mbl, mCherry-MreB appear in lane 2)

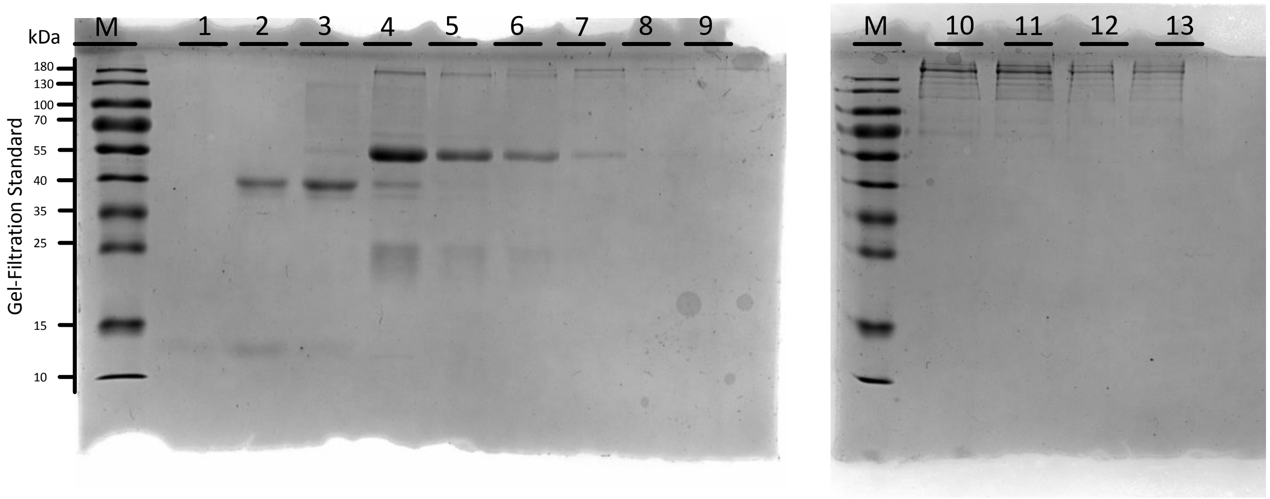

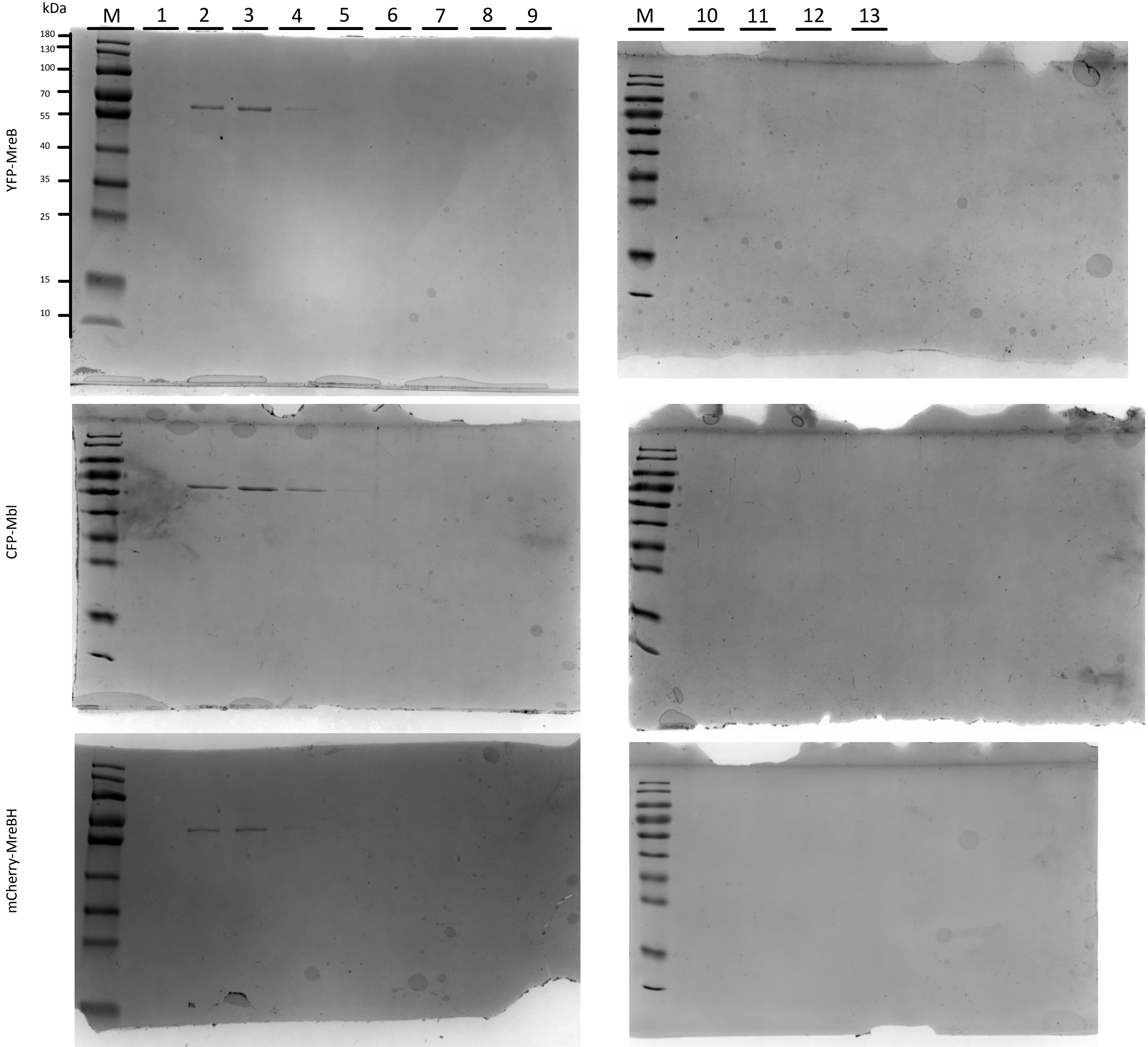

kDa
M
1
2
3
4
5
6
7
8
9
M
10
11
12
13
180
130
100
70
55
40
35
25
15
10
YFP-MreB
CFP-Mbl
mCherry-MreBH

Western blot of isolated YFP-MreB, CFP-Mbl, mCherry-MreBH monomers with specific antibodies against the respective protein (V: void volume, M: isolated monomer peak, S: after dialysis to low salt polymerization buffer), corresponding to figure 1.

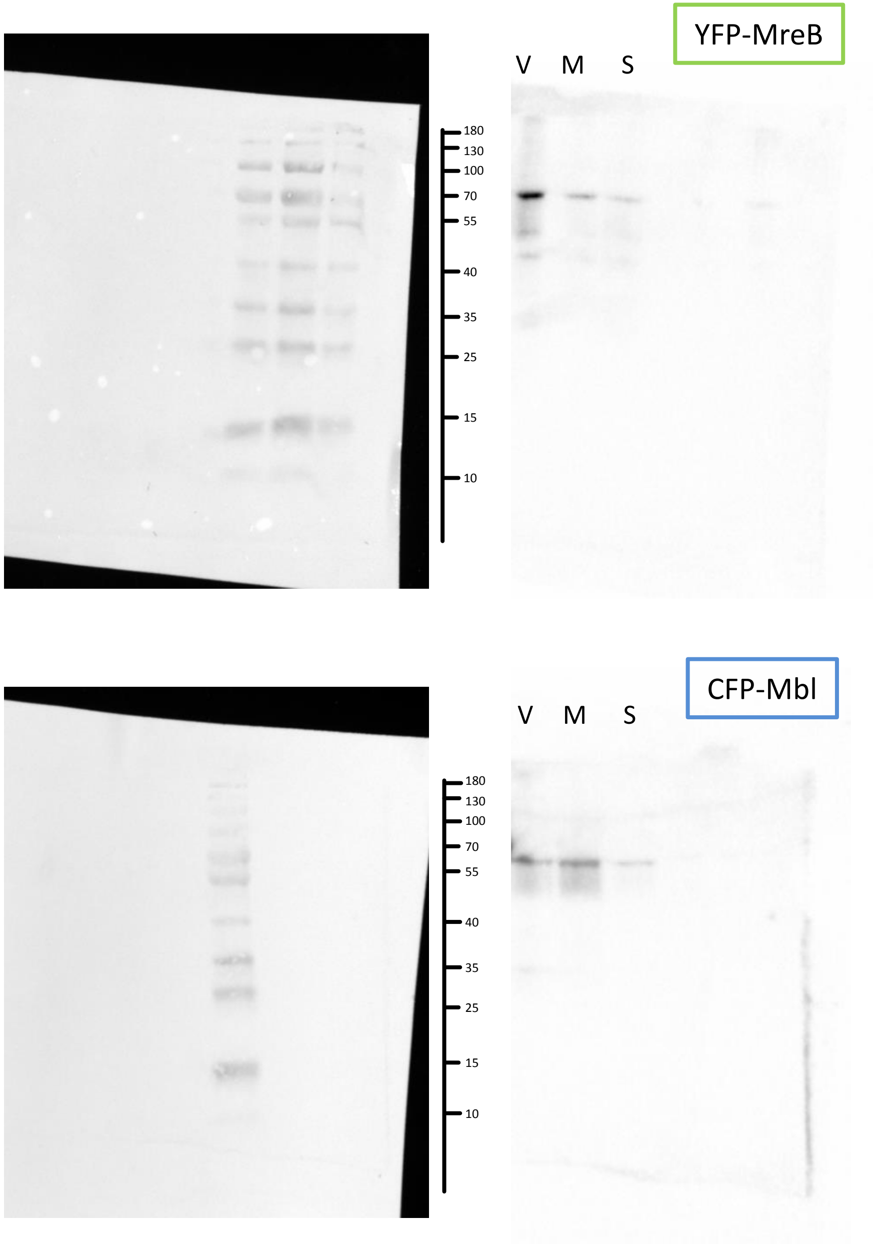

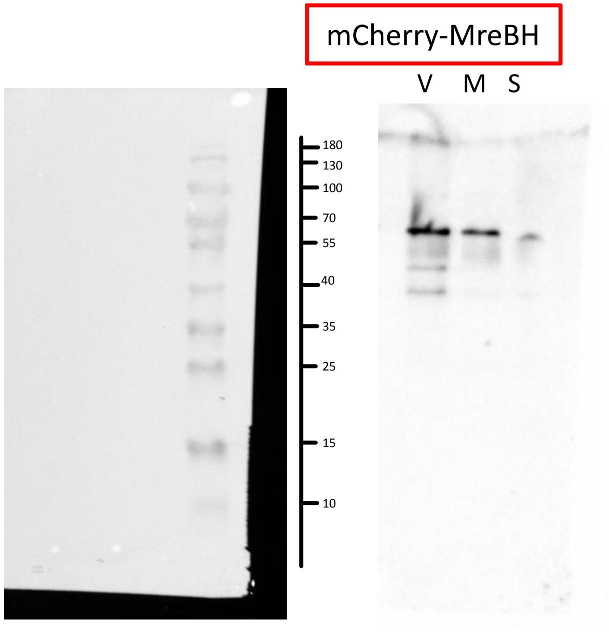

Supplement: Supplementary file 12 — Additional file 12. [file 12860_2020_319_MOESM12_ESM.pdf]
